# Graphite Anodes for Li-Ion Batteries: An Electron Paramagnetic Resonance Investigation

**DOI:** 10.1021/acs.chemmater.3c00860

**Published:** 2023-07-13

**Authors:** Teresa Insinna, Euan N. Bassey, Katharina Märker, Alberto Collauto, Anne-Laure Barra, Clare P. Grey

**Affiliations:** † Yusuf Hamied Department of Chemistry, University of Cambridge, Lensfield Road, Cambridge CB2 1EW, United Kingdom; ‡ Centre for Pulse EPR (PEPR), Imperial College London, London W12 0BZ, United Kingdom; § LNCMI-CNRS, EMFL, Univ. Grenoble-Alpes, 25 Rue des Martyrs, B.P. 166, 38042 Grenoble Cedex 9, France; ∥ LNCMI-CNRS, EMFL, Univ. Toulouse 3, Insa Toulouse, 118 Route de Narbonne 31062 Toulouse Cedex 9, France

## Abstract

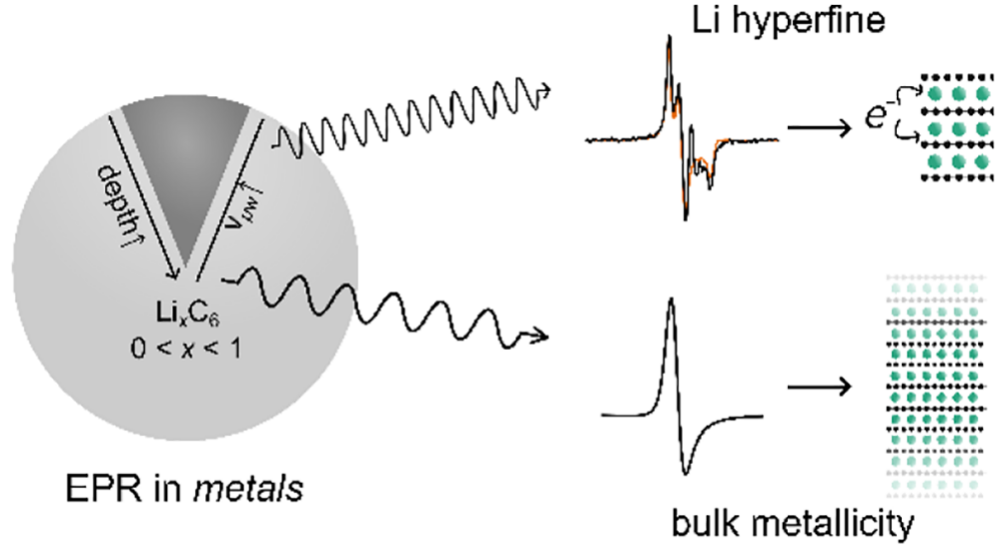

Graphite is the most commercially successful anode material for lithium (Li)-ion batteries: its low cost, low toxicity, and high abundance make it ideally suited for use in batteries for electronic devices, electrified transportation, and grid-based storage. The physical and electrochemical properties of graphite anodes have been thoroughly characterized. However, questions remain regarding their electronic structures and whether the electrons occupy localized states on Li, delocalized states on C, or an admixture of both. In this regard, electron paramagnetic resonance (EPR) spectroscopy is an invaluable tool for characterizing the electronic states generated during electrochemical cycling as it measures the properties of the unpaired electrons in lithiated graphites. In this work, *ex situ* variable-temperature (10–300 K), variable-frequency (9–441 GHz) EPR was carried out to extract the **g** tensors and line widths and understand the effect of metallicity on the observed EPR spectra of electrochemically lithiated graphites at four different states of lithiation. We show that the increased resolution offered by EPR at high frequencies (>300 GHz) enables up to three different electron environments of axial symmetry to be observed, revealing heterogeneity within the graphite particles and the presence of hyperfine coupling to Li nuclei. Importantly, our work demonstrates the power of EPR spectroscopy to investigate the local electronic structure of graphite at different lithiation stages, paving the way for this technique as a tool for screening and investigating novel materials for use in Li-ion batteries.

## Introduction

Graphite is the most widely used anode material for Li-ion batteries, and its low electrochemical potential, low cost, low toxicity, and high abundance make it ideally suited for a variety of applications, such as batteries for devices, transportation, and grid-based storage.^1−4^ It is associated with high capacities (372 mAh g^–1^), which can be retained over many cycles, and is capable of hosting several different alkali metal cations in addition to Li.^5−9^

**Figure 1:**
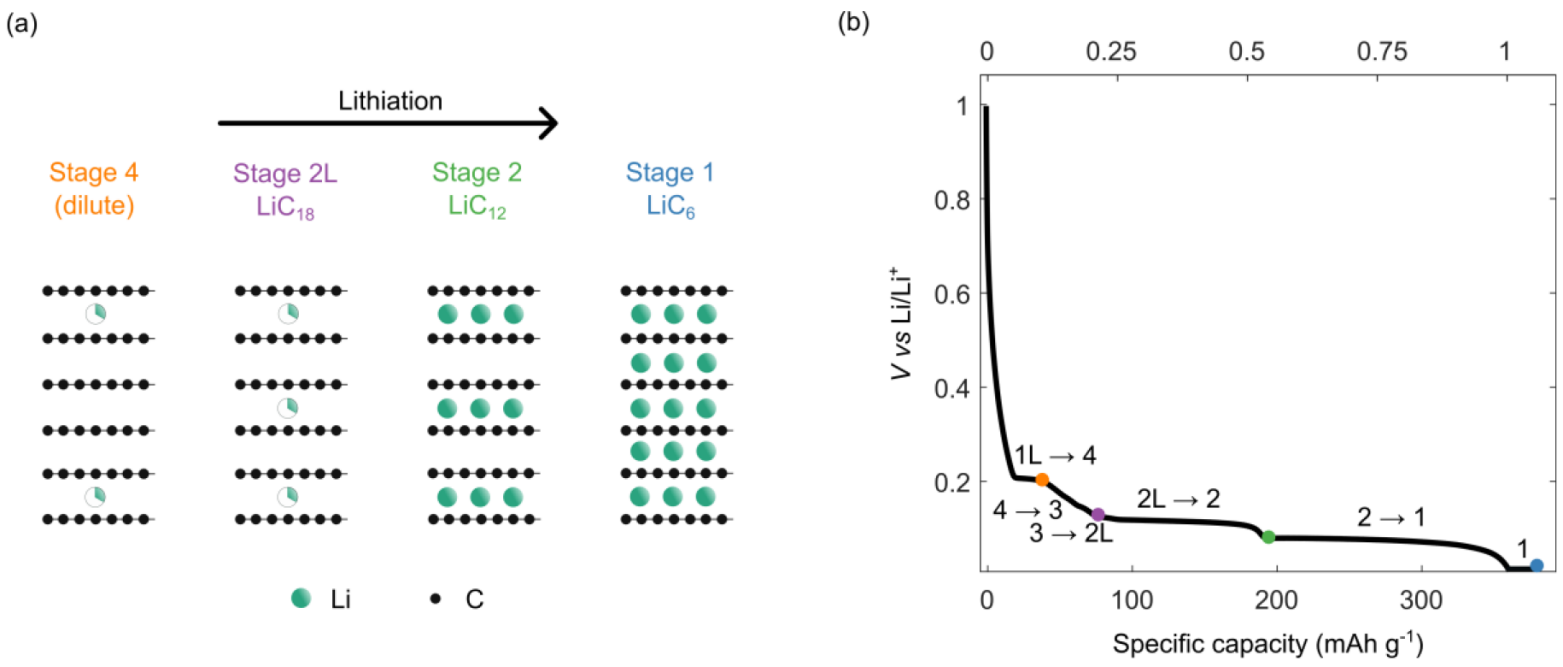
(a) Li intercalation stages as isolated in this work. The dilute stages are formed first (1L, followed by stages 4 and 3, the last two distinguished by the number of graphite sheets between the Li layers) up to a 25% state of charge (where “charge” is used, because graphite is typically charged vs a positive electrode material (cathode)). After this, the dilute stage 2 (2L) and the dense stages 2 and 1 form. The interlayer spacing was kept constant for the sake of clarity in this figure, but as the concentration of Li^+^ ions increases, the *c* lattice parameter, and thus interlayer spacing, increases by ≤10% (see the text). (b) Typical voltage profile for constant-current cycling of a Li:graphite half-cell. The transitions between intercalation compounds are indicated along the voltage profile, and states of charge measured by EPR are indicated with color-coded dots.

A range of mechanisms have been proposed in the literature that describe Li intercalation in graphite.^9−17^ Among these, the Daumas–Hérold mechanism describes the lithiation of graphite as comprising at least four stages,^10^ where the stage number indicates the number of graphite sheets between the Li layers;^9^ for example, stage 1 denotes LiC_6_, comprising alternating layers of Li^+^ ions and graphite sheets (Figure 1a). Stages formed above 0.076 V versus Li/Li^+^ are called “dilute” as only partial occupancy of Li sites between the graphite layers is found, while those formed below that potential are called “dense” (2 and 1, corresponding to LiC_12_ and LiC_6_, respectively), with the occupied Li layers being fully filled by Li.

This mechanism is now considered as simplified. Subsequent work by Dahn^12^ revealed the presence of additional dilute stages at low states of charge (0.04 < *x* < 0.3 in Li_*x*_C_6_), and work by Ohzuku et al.^16^ revealed the existence of a “liquid” (or dilute) stage 1 (1L) prior to stage 4 and an additional stage 2L between stages 3 and 2 (Figure 1b). In these “liquid-like” stages, there is no visible Li ordering in the *a–b* planes.^15^ These results were later confirmed using *in situ*^7^Li nuclear magnetic resonance (NMR).^13^ More recently, *operando* neutron diffraction by Didier and co-workers^15^ confirmed the presence of a hysteresis between lithiation and delithiation of graphite in the dilute stage region.

While the physical and electrochemical properties of graphite have been thoroughly characterized,^15−20^ there are still open questions regarding the electronic structure of graphite. Where are the electrons located in the different stages of lithiated graphite? Do they occupy localized states on Li, delocalized states on C, or an admixture thereof?

Perhaps the most convenient, non-invasive technique for studying unpaired electrons is electron paramagnetic resonance (EPR) spectroscopy, which probes unpaired electron spin microstates in a fashion analogous to nuclear spin microstates in NMR. The method has been applied to study, for example, Li metal dendrites and a number of cathode materials.^21−23^ Here we use high- and low-field EPR to explore the electronic properties of Li-intercalated graphite for battery applications. Our studies were performed on high-performance, battery-grade graphite anodes, with the stages being isolated electrochemically to improve our understanding of graphite as an anode material. The graphite stages were isolated electrochemically by stopping the cells at the voltage cutoff of 0.20 V (nominally stage 4), 0.12 V (stage 2L), 0.076 V (dense stage 2), and 0.005 V (dense stage 1) versus Li/Li^+^. Pulsed EPR (PEPR) was then used to measure electron relaxation times, while high-frequency (HF) EPR allowed us to increase our signal resolution.

Unlike continuous wave (cw) EPR, where the electron spins microstates are excited under constant irradiation by microwaves, pulsed EPR applies nanosecond-long microwave pulses to excite a region of the spectrum in manner similar to conventional NMR spectroscopy, the difference being that the frequencies used here are on the order of gigahertz (not megahertz) and that the pulse lengths are 3 orders of magnitude shorter (i.e., nanoseconds) than in NMR due to the faster relaxation times of electron spins and the need to excite a broader bandwidth. In contrast, HFEPR (performed in cw mode as it is more challenging experimentally to generate pulses at high frequencies) is a powerful technique for increasing spectral resolution.

In the context of battery anode production, graphite is incorporated into electrodes together with other components, among them Super Porous (SuperP) carbon or carbon black. These components are typically added to increase the electrical conductivity within the electrode so that it can sustain faster cycling rates.^24,25^ However, they are also EPR active, and their signals overlap with those of the active material, altering the signal position, line shape, and line width. This work examines these components individually to separate their signals from those of graphite.

We show that combining cw and pulsed EPR at variable temperatures (VT) and frequencies provides a more holistic picture of graphite’s electronic properties. Lower frequencies (∼9 GHz, X-band) aid in the understanding of the bulk metallic structure of Li-intercalated graphite, while higher frequencies provide a unique handle on the local electronic structure. We support our spectroscopic data with magnetometry to aid interpretation of the magnetic interactions present in these materials.

We begin this paper with a brief overview of EPR methodology as applied to metals, as EPR spectroscopy of metallic phases is less well described in recent literature; an understanding will be of particular importance for lithiated graphite.^26,27^ We then provide a short overview of the EPR and electronic conductivity literature on pristine graphite and graphite intercalation compounds.

## Background

### Electron Paramagnetic Resonance (EPR)

In general, electron spin microstates are degenerate in the absence of a magnetic field but become nondegenerate under an applied magnetic field, **B**, due to the electron Zeeman interaction:
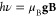
1where *h* is Planck’s constant, ν the incident microwave frequency, μ_B_ the Bohr magneton, and **g** the **g** tensor, a second-rank tensor that is diagnostic of the local environment of unpaired electrons.

The principal components of the **g** tensor reflect both the influence of spin–orbit coupling (SOC) on the energies of the electron spin microstates and the nature of the orbitals that contain unpaired spin density:
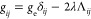
2where the first term describes the Zeeman splitting for a free electron (i.e., one that does not interact with its surroundings), *g*_e_ is the free electron *g* factor (2.0023), and δ_*ij*_ is the Kronecker delta. The second term represents the SOC component of the *g* factor, with λ being the single-electron SOC constant and Λ_*ij*_ the integral representing overlap between the ground and excited states that are coupled by the SOC interaction.^28,29^ This second term is known as the *g* shift, and its value is inversely proportional to the energy difference between the ground and excited states being coupled via the SOC interaction. For strongly localized paramagnetic centers, a more than half-filled shell of electrons results in a negative SOC contribution, and therefore, *g* > *g*_e_; a less than half-filled shell results in a positive contribution of the SOC and *g* < *g*_e_.^30^ In systems with delocalized orbitals, the *g* factor depends on SOC, band occupancy, and the presence of defects and dopants.^31^ It should be noted that a small change in *g* represents a very large difference in the resonance position. At X-band frequencies, a change in *g* of 0.01 corresponds to a difference in field position of 2 mT. Through the *g* factor, EPR can thus provide useful information about the electronic structure (via the SOC component of the **g** tensor) and the symmetry of the orbital containing the unpaired spin (isotropic, axial, or rhombic).

**Figure 2:**
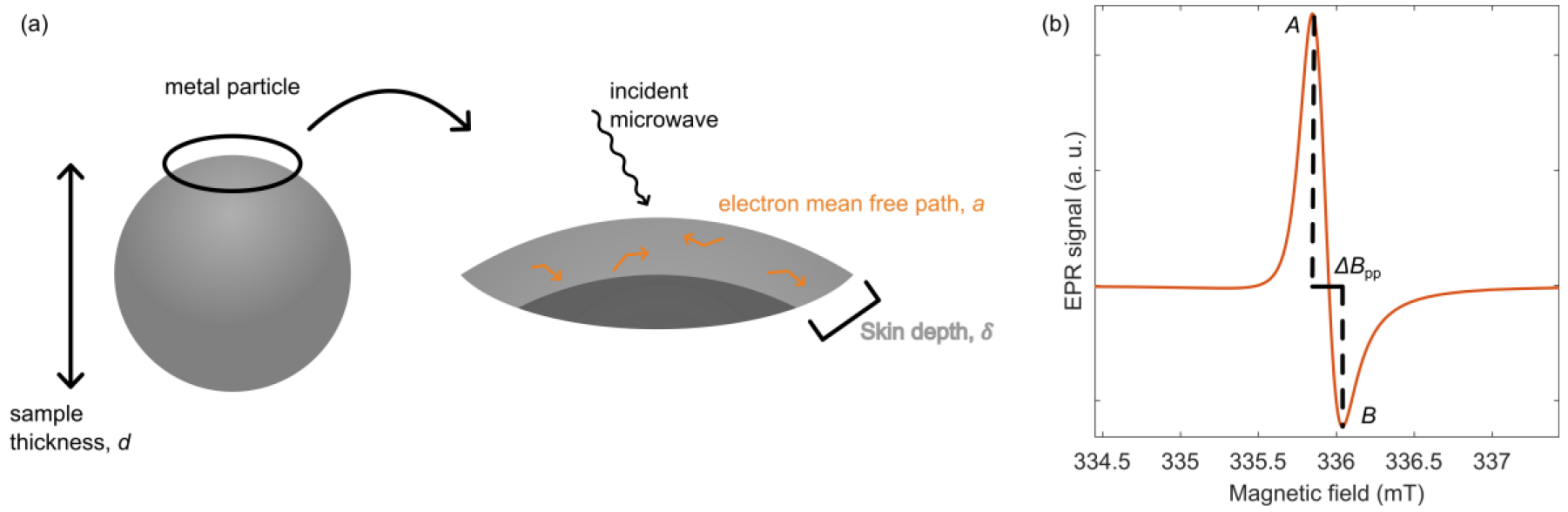
(a) Illustration of the skin depth of a generic metal particle with diameter *d* upon incoming microwave radiation. (b) Typical EPR cw line shape of conduction electrons in a metallic system. The intensity of the positive peak, *A*, is larger than the intensity of the negative peak, *B*, resulting in a Dysonian line shape. The peak-to-peak line width, *ΔB*_pp_, is also defined.

The ability to excite conduction electrons in a metal is often weakened severely (Figure 2a). Conduction electrons attenuate the microwaves, a phenomenon known as the skin effect, also causing phase shifts of the incident microwaves, with the distance into the bulk at which the amplitude of the microwave field is reduced by a factor of 1/*e* being termed the skin depth, δ:^26,32^
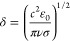
3where *c* is the speed of light, ε_0_ is the vacuum permittivity, σ is the electrical conductivity of the metal, and ν is the applied microwave frequency. Only conduction electrons passing through the skin depth are irradiated. Hence, the spin concentration of a metallic sample cannot be simply determined from the double integral of the spectrum, unlike in systems with localized electrons.^33^ For LiC_6_, at X-band frequencies (∼9 GHz), δ ∼ 1 μm (assuming an electrical conductivity of 2.4 × 10^5^ S cm^–1^, measured for transport in the *a–b* plane at room temperature),^6^ while many graphitic particles are many micrometers in size [Hitachi MagE3 graphite has an average particle size of 13 μm (Figure S1)]. At the higher frequency of 331 GHz also used in this study, δ is even smaller, decreasing to ∼0.5 μm. The anisotropy in conduction and thus the skin depth are discussed in more detail below.

Skin effects can be classical or anomalous depending on the relative magnitudes of δ and the electron mean free path, *a*, i.e., the distance the electron travels before a collision with a defect in the lattice or with a lattice phonon occurs, with the scattering process changing its velocity (and thus direction of travel). δ > *a* defines the classical scenario, and δ < *a* the anomalous scenario, where electrons can travel in and out of the skin depth region multiple times before a scattering process occurs.^32^ Anomalous skin effects are more likely as the temperature is decreased, or the impurity concentrations are reduced as *a* increases.

Metallic systems often exhibit asymmetric EPR resonances (Figure 2b), potentially providing further valuable information about the skin depth and thus the degree of metallicity. In the derivative spectrum, the asymmetry is quantified via an asymmetry parameter *A*/*B* > 1, where *A* and *B* correspond to the intensities of the positive and negative peaks, respectively (in isotropic, nonmetallic systems, *A*/*B* = 1). This asymmetric line shape is a consequence of both the fast movement of the electrons and the skin effect (Figure 2a), the electrons moving in and out of the skin depth region during cw irradiation, experiencing cw irradiation at random intervals. The phase-shifted EPR line shapes of metallic samples (Figure 2b) were modeled theoretically by Dyson^26^ and then first observed experimentally by Feher and Kip.^27^ Dyson considered a classical skin effect and modeled the line shapes as a function of skin depth, δ, and sample thickness. The thickness/skin depth ratio, *d*/δ, determines the *A*/*B* ratio and the contribution of absorptive and dispersive components to the observed EPR line shape. The EPR line shape of a very thin metallic film (i.e., *d* ≪ δ) has a dominant absorptive component (and is more symmetric), whereas that of a thick metallic film (*d* ≫ δ) has a dominant dispersive component.^34^

A full analysis needs to consider multiple factors, including the geometry of the metal particles and the spin depth, i.e., the distance an electron travels between spin flips; this distance is generally greater than the average distance between collisions, *a* (i.e., the mean free path of the electron), because not all collisions result in a change in spin. While a number of approaches have been taken to model these systems,^32^ an important factor is the *T*_D_/*T*_2e_ ratio (where *T*_2e_ is the transverse relaxation time of the electron or spin lifetime and *T*_D_ is the diffusion lifetime, defined as the average time taken for an electron to move in and out of the skin depth). *T*_D_ is a function of the skin depth, the velocity of the electron at the Fermi level, ν_F_, and the mean free path of the electron, *a*
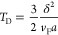
4*T*_D_ can be also expressed in terms of skin depth (and thus conductivity) and electron diffusivity, *D*(32,35)
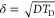
5It should be noted that *T*_2e_ still sets the line width of the resonance, even if *T*_D_ is shorter than *T*_2e_, since the conduction electrons can, in principle, leave and return to the skin depth region multiple times during time interval *T*_2e_. As the skin depth decreases, the *A*/*B* ratio increases, reaching a maximum that depends on the *T*_D_/*T*_2e_ ratio. For spherical particles and when *T*_2e_ ≫ *T*_D_, extremely large values of *A*/*B* can result when *d*/δ is large. For small values of *d*/δ, however, the *A*/*B* ratio is essentially independent of *T*_D_/*T*_2e_ (see Figure 15 of ref (32)).

### EPR and Conductivity Studies of Graphite

EPR studies of pristine graphite were conducted in the 1950s and 1960s.^36−39^ The first spectrum was recorded by Castle^36^ and contained a signal centered at *g* = 2.0083. A few years later, Wagoner carried out a more exhaustive EPR study on single crystals of graphite with Cu-covered edges to remove the signal from dangling bonds and isolate the signal coming from conduction electrons largely moving in the *a–b* plane.^37^ The temperature dependence of the intensity was linked to Pauli paramagnetism, rather than Curie–Weiss paramagnetism, as the signal intensity increased linearly with temperature (which is analogous to polycrystalline graphite).^38^ Additionally, the deviation of the *g* factor from the free electron *g* value (the *g* shift) was dependent on temperature, with smaller *g* shifts at higher temperatures; a large *g-*anisotropy was observed, with *g*_⊥_ remaining constant with temperature but *g*_||_ being strongly temperature dependent. This was explained by the increasing thermal population of states away from the band edge, which have smaller values of *g*_||_. This result agrees with theoretical work by McClure, which was based on band structure calculations and the position of the Fermi level.^39^ Dresselhaus and co-workers performed a more detailed analysis of the effect of the SOC on the degeneracies of the π bands near the Fermi energy; holes and electrons are present at the Fermi level of graphite, and the *g* shift arising from an average of all the contributions from electron and hole transitions at the Fermi surface. Wagoner also showed that impurities result in variations in the *g* shift. For example, upon addition of boron atoms, the *g* shift decreased with an increase in B concentration.^37^ This finding is counterintuitive on the basis of simple molecular orbital theory arguments, as one would expect the *g* shift to increase at higher B contents (due to the addition of holes to the bands). The *g* shift was tentatively ascribed to the lifting of the degeneracy of the two π bands in graphite now via the substitution of B on one of the carbon sublattices.^39^ Narrower line shapes were seen for graphite at higher temperatures, line broadening being ascribed to a spin-lattice (*T*_1e_) relaxation process involving scattering (spin-orbit coupling) to impurity atoms.^40^

The results obtained for single-crystal graphite were subsequently confirmed also in polycrystalline samples,^38^ whose EPR signature no longer revealed a noticeable *g* anisotropy, which was ascribed to the electron mobility, the electrons moving through a large number of different crystallites with different orientations on the timescale of the *T*_1e_, resulting in motional averaging.^37^

The electrical conductivity of graphite in the *a* (*b*)- and *c*-directions, σ_*ab*_ and σ_*c*_, respectively, is substantially increased as Li^+^ ions are intercalated, electrochemically or chemically.^7^ This is due to an increase in the number of charge carriers (introduced by reduction of graphite) and number of Li^+^ ions between the layers.^8^ While it is intuitive that these additional charge carriers improve the *ab* conductivity upon lithiation, understanding how the overall conductivity anisotropy is affected is less obvious. The latter is defined as σ_*ab*_/σ_*c*_, with values of >1 indicating preferential movement of electrons within the layers and values of <1 that between the layers. This ratio is considerably lower in Li-intercalated graphite than in other alkali–graphite intercalation compounds, a consequence of the small size of Li^+^ ions. The interlayer spacing increases by only ∼10% upon lithiation,^4^ and therefore, the electrons introduced into the π* orbitals in the *a–b* planes are not fully decoupled from the adjacent layers.^6^ Additionally, the intercalated Li^+^ ions are thought to be partially involved in facilitating electrical conductivity in the *c*-direction via overlap of the C 2p_*z*_ orbitals with the Li 2s orbitals.^4,5,41^

If the particle size or sample thickness of graphite is larger than its skin depth (i.e. *d* ≫ δ), the increase in the overall conductivity upon lithiation results in a decrease in the skin depth and consequently increased line shape distortions in EPR spectra.^26,27,32^ On a more practical note, the increasing electrical conductivity upon charging is one of the reasons for the success of graphite in a lithium-ion battery allowing moderately high charging rates.^24^

In the 1970s and 1980s, graphite intercalation compounds were of particular interest due to their potential as superconductors, resulting in a variety of studies that assessed their physical properties and the tunability of the Fermi level via intercalating donors (such as Li, K, Rb, and Cs) and acceptors (such as HNO_3_, H_2_SO_4_, and AsF_5_).^6,7,42^ The increasing donor size results in greater interlayer spacing as well as an increase in SOC and therefore electron relaxation rate (confirmed by broader EPR line widths).^43,44^ To the best of our knowledge, the only EPR study that covered at least three different lithiation stages of graphite was carried out by Lauginie and co-workers,^42^ who compared the electronic properties of a range of HOPG donor and acceptor graphite intercalation compounds. This work builds on their study, extending to more disordered carbons and exploring the use of high-field and pulsed EPR spectroscopy.

## Experimental Section

### Materials

Graphite electrodes were fabricated in the Argonne National Laboratory Cell Analysis, Modeling and Prototyping (CAMP) facility and are composed of 91.83 wt % graphite powder (Hitachi MagE3), 2 wt % carbon black (Timcal C45), 6 wt % PVDF binder (Kureha 9300), and 0.17 wt % oxalic acid; this mixture is coated on Cu foil. The areal loading of the graphite active material is 5.83 mg cm^–2^. The graphite electrodes were punched out into discs (15 mm diameter), dried under vacuum at 120 °C for 12 h, and then transferred to an Ar glovebox without being exposed to air. The electrolyte used in this study was LP30 [1.0 M LiPF_6_ in ethylene carbonate, 1:1 (v/v) EC/dimethyl carbonate (DMC), battery-grade, Sigma-Aldrich]. Li metal discs were purchased from LTS Research Laboratories, Inc. The separators were glass microfiber (Whatman GF/B) slices cut into 16 mm disks. All procedures described below (coin cell assembly and disassembly and EPR capillary preparation) were performed in an Ar-filled glovebox with water and oxygen levels of <5 ppm.

### Electrochemistry

All electrochemical tests were performed in graphite/Li half-cells in 2032 coin cells (Cambridge Energy Solutions). One graphite electrode disc, a glass fiber separator soaked with 100 μL of the LP30 electrolyte, and one Li metal disc were stacked and assembled into the coin cell.

The cells were cycled galvanostatically [constant-current constant-voltage (CCCV) cycling] at room temperature (20 ± 2 °C) on a VMP2 potentiostat (Biologic) at a rate of *C*/20 assuming a graphite capacity of 360 mAh g^–1^. All potentials reported in this work are referenced against Li/Li^+^.

The four different graphite stages were isolated after cycling the cells for two conditioning cycles at *C*/20 and then charging (lithiating) to a voltage cutoff of 0.20 V for stage 4, 0.12 V for stage 2L, 0.076 V for dense stage 2, and 0.005 V for dense stage 1. The cells were opened in a glovebox, and the graphite electrodes were extracted, rinsed with DMC, and dried under dynamic vacuum for 15 min to remove excess solvent. The electrode materials were scraped off the Cu current collector, packed in quartz capillaries (1 mm diameter, Bruker), and sealed (two-component epoxy) for EPR characterization.

### Solid-State NMR (ssNMR)

The phase purity and the extent of lithiation were verified by ^7^Li ssNMR. ssNMR spectra were acquired on an 11.74 T Bruker NMR spectrometer. Samples were packed under Ar from cycled electrodes into 1.3 mm ZrO_2_ outer diameter (o.d.) rotors. The magic angle spinning (MAS) frequencies used were 30 kHz for the dense stages and 40 kHz for the dilute stages, and spinning was performed under N_2_. Radiofrequency (rf) pulses used a 125 kHz field strength for the dilute stages and a 104 kHz field strength for the dense stages, with the pulse length being calibrated on the sample due to metallicity. The chemical shift was referenced to solid LiF (−1.00 ppm). The spectra are shown in Figure S2.

### X-Band Continuous Wave EPR Spectroscopy

*Ex situ* cw EPR spectroscopy was carried out on an X-band benchtop EPR spectrometer (E5000, Magnettech) set at a microwave frequency of 9.477 GHz and equipped with a variable-temperature (VT) unit connected to the cavity. The samples were loosely packed into low-background 1 mm o.d. glass capillaries, although in one case (where noted), the sample packed in the NMR rotor was measured. A modulation field of 0.1 mT was applied at a 100 kHz modulation frequency. Except where otherwise stated, a microwave power of 5 mW was applied. VT experiments over the temperature range of 300 to 100 K had a 10 K step between measurements, and the temperature was allowed to equilibrate for 1 min before measurement. The *g* factor was calibrated using a Mn^2+^ in ZnS standard.

Due to the Dysonian line shape of the resonances, the *g* factor cannot simply be read from the point at which the spectrum passes through zero intensity; instead, it was analytically extracted from the field corresponding to the center of gravity of the spectrum.^32^

### High-Frequency cw VT EPR Spectroscopy

High-frequency (HF) cw EPR spectra were recorded on a double-pass transmission EPR spectrometer at the Laboratoire National des Champs Magnétiques Intenses (LNCMI, Grenoble, France).^45,46^ The frequencies were varied from 331 to 441 GHz using two frequency sources (one operating at 127 GHz and the other at 110 GHz) and their multipliers, while detection was achieved using a bolometer. Temperatures were recorded by using a VT insert (Cryogenic). The spectra were recorded at 10, 25, 50, and 140–170 K at the sweep rates reported in Table S1. The graphite powders were packed in 4 mm o.d. quartz tubes (Wilmad, Sigma-Aldrich), and the powders were covered in nonane (99%, anhydrous, Sigma-Aldrich) to prevent them from experiencing torquing effects (i.e., movement of the powders driven by the anisotropic susceptibility). The tubes were sealed using epoxy glue in an Ar-filled glovebox. Phase correction of the HFPER spectra was achieved through an in-house processing script, using spherical harmonics. The EPR spectra were fitted to a powder pattern line shape with anisotropic *g* using the EasySpin toolbox for MATLAB.^47^ The fitted **g** tensors were then corrected by using a field calibration factor obtained from a control sample (Mn^2+^ in MgO), as described further in Figure S3.^45^ A phase fitting parameter in Easyspin was used to account for phasing caused by skin depth.

### Pulsed EPR Spectroscopy

The pulsed EPR measurements were performed at Imperial College London’s Centre for Pulse EPR (PEPR) using a Bruker Elexsys E580 X-band EPR spectrometer equipped with a 5 mm split-ring resonator (Bruker, model ER4118X-MS5-W) and a 1 kW TWT amplifier (Applied Systems Engineering Inc., model 117X). Temperature control was achieved using a closed-circuit cryostat (Cryogenic Ltd.) controlled by a Lake Shore 350 ITC (Lake Sho Cryotronics, Inc.).

FID-detected long pulse saturation recovery traces were recorded using the sat–*t*–π/2–acq pulse sequence, whereby the length of the π/2 pulse was set to 16 ns and the saturation (sat) pulse was set to 1 μs; all pulses had the same amplitude. A complete 16-step phase cycle (cf. Table S2) was used to suppress unwanted signals. The interpulse delay *t* was incremented in steps of 10 ns starting from a minimum value of 10 ns between the falling edge of the saturation pulse and the raising edge of the π/2 detection pulse. For each *t* value, the whole FID transient was acquired with a 1.0 ns time resolution of the digitizer (Bruker, model SpecJet-III); the transients were integrated afterward to yield the recovery traces, and the extracted intensities were then fit to extract an apparent *T*_1e_. The *T*_1e_ values were also measured using an inversion recovery (π–*t*–π/2–acq) sequence, and similar values were obtained.

All pulse experiments were performed at the maximum of the EPR line. The repetition time was set to 100 μs (FID) or 200 μs (saturation recovery). The microwave pulses were generated using the SpinJet AWG unit (Bruker); the individual pulse phases (+*x*, +*y*, −*x*, −*y*) were calibrated at each temperature (Table S2).

## Results

### Pristine Graphite and Battery-Grade Carbons

### cw X-Band VT EPR Spectroscopy

**Figure 3:**
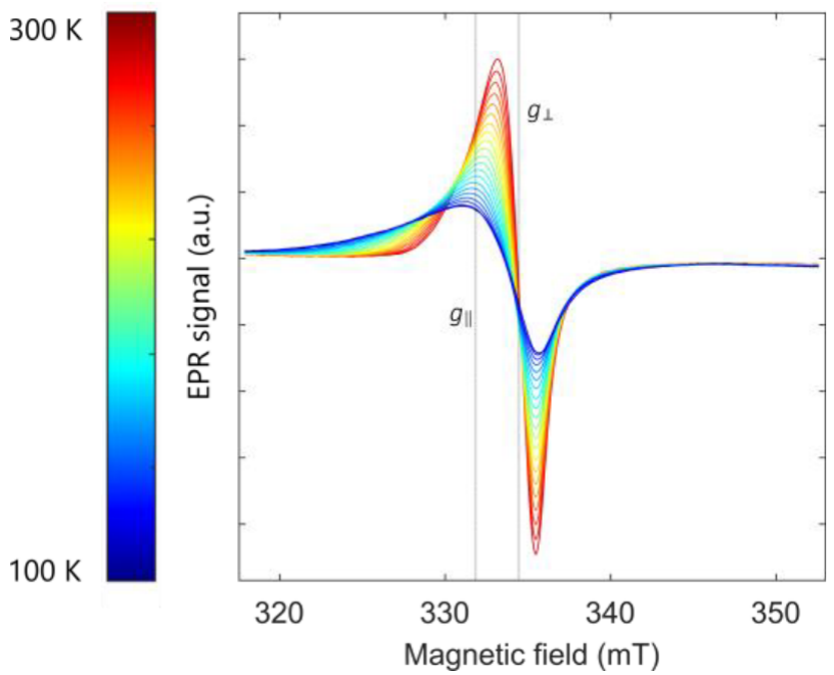
Variable-temperature X-band EPR spectra of pristine Hitachi MagE3 graphite taken from 100 K (blue) to 300 K (red) in temperature steps of 10 K at a microwave power of 1 mW. The line width increases as the temperature decreases, and the *g_||_* component moves to higher *g* values and a lower magnetic field. The *g* values measured at 300 K are marked.

We begin with a brief examination of the pristine graphite material. The room-temperature X-band EPR spectrum of pristine graphite (Hitachi MagE3) contains a single line at *g* = 2.0319 (Figure 3, red trace). The signal is attributed to thermally excited electrons in the conduction band and/or defect spins from surface dangling bonds.^31,37^ Because of the axially symmetric crystal structure (*a* = *b* = 2.462 Å, and *c* = 6.720 Å)^48^ and two-dimensional band structure, it is anticipated that unpaired electrons in graphite would give rise to an axial signal. Discontinuities are likely not clearly resolved (i.e., no separate *xy* and *z* components are seen) because of the low frequencies used at X-band, resulting in significant overlap of the components of the **g** tensor, *g*_||_ and *g*_⊥_, corresponding to components in and out of the plane, respectively, of the graphite layers.^31^ On cooling from 300 to 100 K, the signal broadens (from a peak-to-peak line width of 0.8 mT at 300 K to 2.4 mT at 100 K), which is at least in part ascribed to the reduction in the extent of motional averaging of signals from the different environments at low temperatures^40^ but also the pronounced shift in *g*_||_ with temperature. The spectra were fitted by including both *g*_||_ and *g*_⊥_ (Figure S4), the *g*_||_ component shifting to higher *g*, from 2.04 ± 0.02 at 300 K to 2.07 ± 0.05 at 100 K (the error is obtained from the *g* strain used to fit the resonances), as observed by others;^37,38,49^*g*_⊥_ remains around 2.02 (2.023 ± 0.006 at 300 K to 2.025 ± 0.005 at 100 K).

The EPR profiles of the conductive carbon species used in electrode fabrication are depicted in Figure S5. Briefly, carbon black has an axial signal (*g*_||_ = 2.04 ± 0.02, and *g*_⊥_ = 2.0182 ± 0.0001), while SuperP carbon is isotropic (*g*_iso_ = 2.0154), the signal likely containing a significant contribution from defect spins within the carbon framework. The signals of these carbon additives are also shifted to higher fields compared to those of graphite, consistent with the observation that more electronically conductive compounds with partially filled conduction bands result in a higher *g* factor.^31^ In the electrodes used in this work, conductive carbon is present at a concentration of 2 wt % (cf. Experimental Section). Given the very low EPR signal intensity per mass and the fact that we are not aiming to extract quantitative information concerning the number of electron spins from our samples, the contribution of these electrode components to the battery anodes was not considered further in this work.

**Figure 4:**
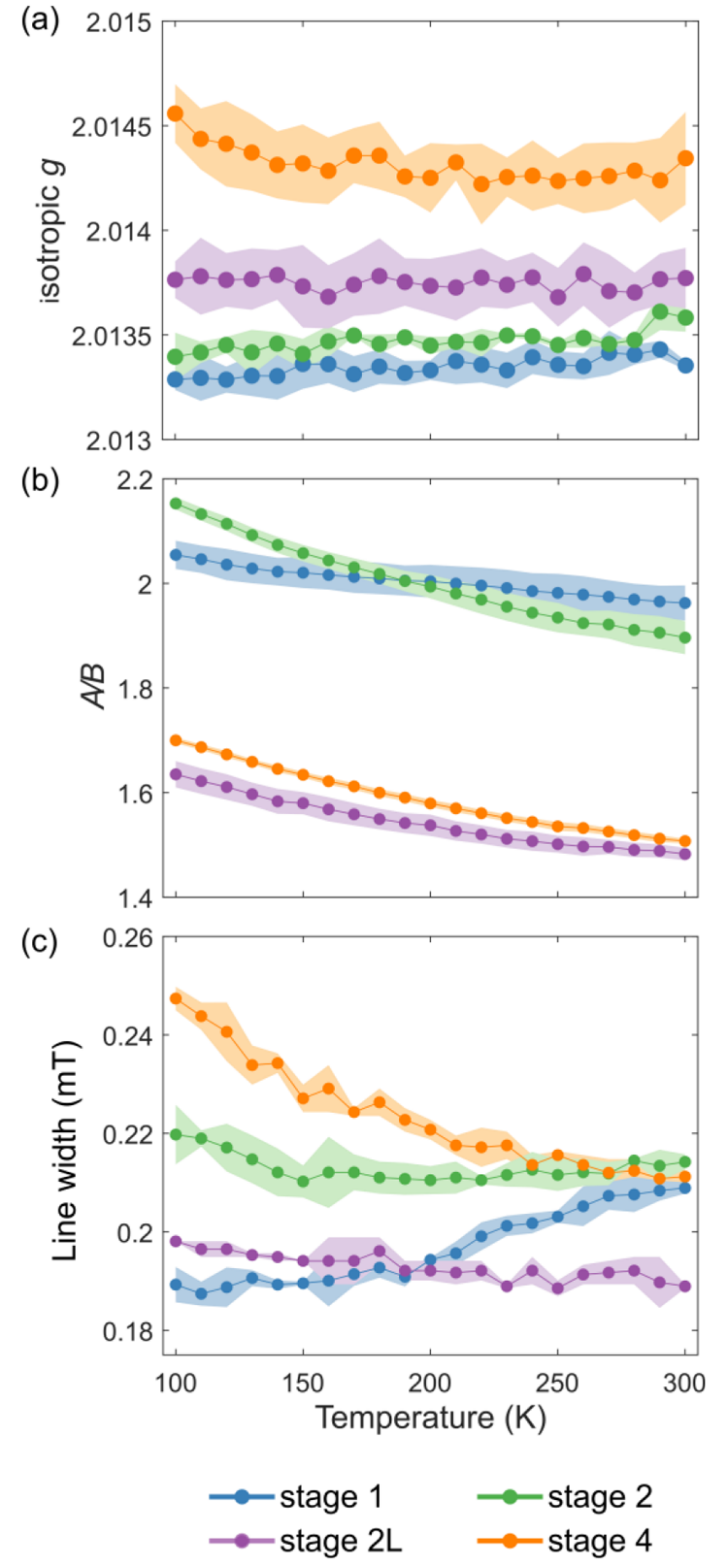
Temperature dependence of the (a) isotropic *g* factors, (b) *A*/*B* ratios, and (c) peak-to-peak line widths for the four lithiation stages of graphite. These parameters were extracted from resonances obtained at X-band in the temperature range of 100–300 K with a temperature increment of 10 K. The shaded area represents errors derived by taking the range of values extracted across four separate samples per lithiation stage.

Variable-temperature X-band EPR of the four graphite stages was performed, and an example of a VT series is shown in Figure S6 (for stage 2); the X-band spectra at 100 K of the four stages are shown in Figure S7. Upon lithiation of graphite (i.e., intercalation of Li^+^ ions and addition of electrons to the conduction band of graphite), the *g* factor shifts toward *g*_e_, from 2.0319 ± 0.0005 (graphite) to 2.0144 ± 0.0003 for stage 4 to 2.0133 ± 0.0001 for stage 1 (Figure 4a and Figure S5) at 300 K as a consequence of the greater contribution of the Li 2s band to the Fermi level and therefore a decrease in the SOC component. The *g* factor remains approximately constant across the temperature range of 100–300 K for all states of charge (Figure 4a), consistent with the population of ground and excited states being temperature-independent around the Fermi level in metals.^32^ Only isotropic *g* factors were extracted for these resonances due to anisotropy not being resolved at these lower frequencies.

For all states of charge, the asymmetry parameter *A*/*B* was higher at lower temperatures (Figure 4b), consistent with metallic systems in which the electrical conductivity increases with a decrease in temperature, leading to a decreased skin depth (eq 3). For the same sample temperature, the values of *A*/*B* indicate that stages 1 and 2 are the most metallic, while stages 2L and 4 are less metallic. We note that stage 2 has a higher *A*/*B* ratio (i.e., is more metallic) than stage 1 below 180 K. We ascribe this to the decrease in interlayer spacing (as the temperature is decreased, as seen for pristine graphite),^50^ the degree of layer collapse being greater in stage 2 due to the presence of alternating Li-deficient layers. We note that care should be taken in comparing *A*/*B* ratios between samples, as these values are also dependent on how densely packed the samples are. For example, a sample of LiC_6_ tightly packed in an NMR rotor gave rise to a signal with an *A*/*B* ratio of 3.80 [vs 1.96, as measured in the more loosely packed capillary (see Figure S8)], at room temperature. Thus, the skin depth and thus ability to excite the bulk sample are a function of not only the individual particle size but also the extent of agglomeration of particles.

The line widths of EPR signals can provide insight into a series of effects, principally *T*_2e_/*T*_1e_ relaxation, the distribution of environments in the sample, and electron mobility.^30^ For stage 4, the line width obeys a temperature dependence similar to that of pristine graphite, where the line width becomes sharper as the temperature increases (Figure 4c). A similar trend is seen for stage 2L; however, the trend is noticeably less pronounced, and the line widths are considerably smaller [at 100 K, ∼0.2 mT in stage 2L compared to 0.25 mT in stage 4 (Figure 4c)]. ^7^Li NMR measurements indicate higher Li^+^ mobility in stage 2L than in dense stages,^51^ the EPR results similarly suggesting motional averaging of the electronic environments as the temperature is increased. The trend is the opposite for stage 1, the line width, somewhat surprisingly, decreasing with temperature and plateauing at ∼150 K. Stage 2 shows a more complex trend in which the line width initially decreases very slightly on cooling but then increases more noticeably, the turnover occurring at 150 K. The increase in line width at low temperatures is tentatively assigned to the onset of magnetic exchange interactions (that become more important at low temperatures), as discussed below.

**Pulsed EPR.** Overall, determining the relative contributions of *T*_2e_ relaxation, distribution of environments, and electronic mobility to the line width is challenging but can be helped by using pulsed EPR measurements. Unfortunately, only the measurements of the longitudinal relaxation times, *T*_1e_, were successful in these systems. Measuring the transverse relaxation times, *T*_2e_, requires a spin–echo sequence, where a delay is applied between the 90° and 180° pulses prior to acquisition. If the *T*_2e_ values are very short, as observed here, the signal is direly within the dead time of the spectrometer and cannot be detected.^52^

**Figure 5:**
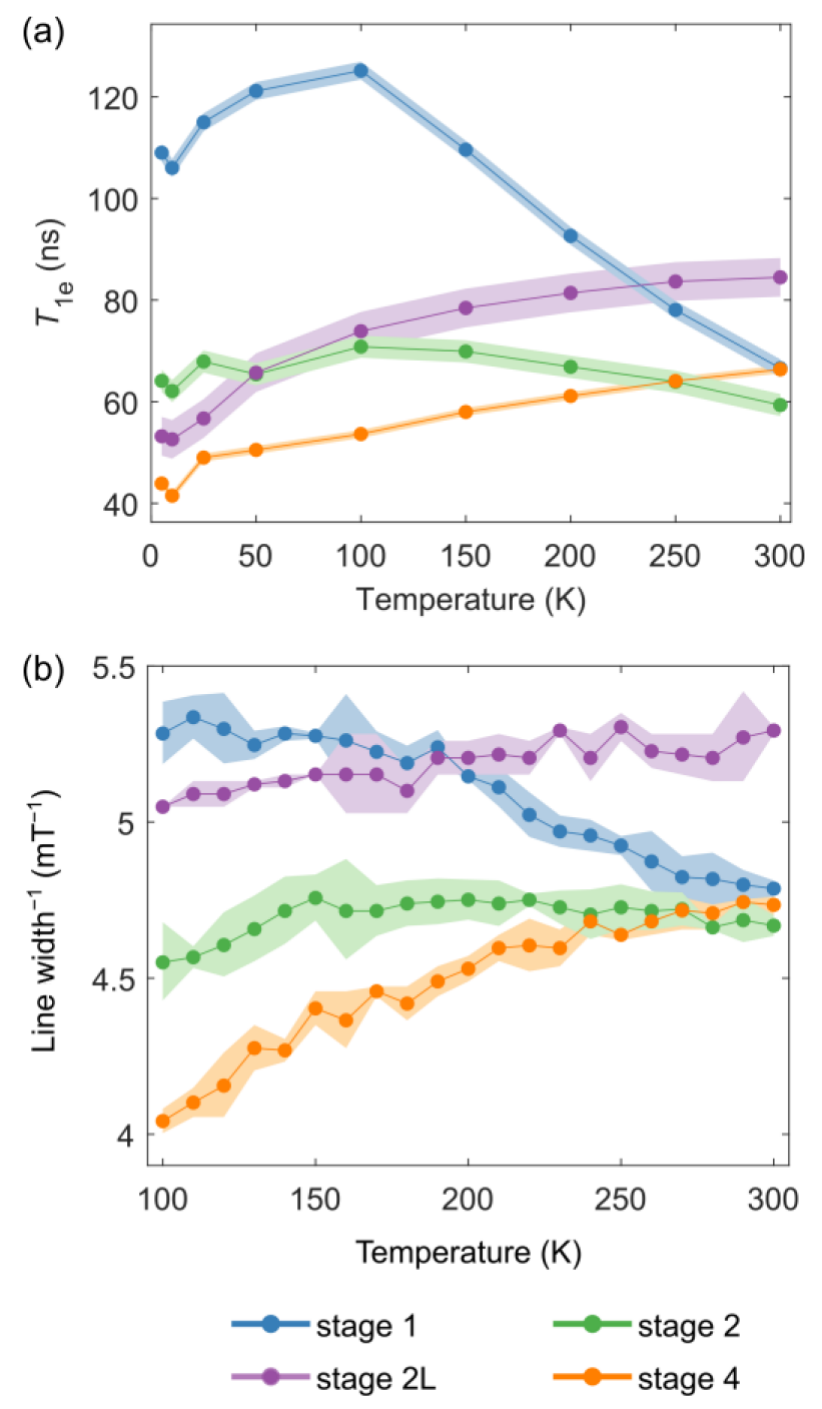
(a) Temperature dependence of the electron longitudinal relaxation times (*T*_1e_) as measured by pulsed EPR for the four lithiation stages of graphite between 10 and 300 K. Two different trends for the dilute and the dense stages are seen, with the former increasing with temperature and the latter peaking at approximately 100 K to then decrease as the temperature increases. (b) Inverse of the line width (reported in Figure 4c) for the four lithiation stages of graphite as measured by cw EPR in the range of 100–300 K. The line widths are strongly affected by *T*_1e_.

The *T*_1e_ values for the four different stages were measured in the temperature range of 10–300 K (Figure 5a) at X-band. For all stages, *T*_1e_ increases as the temperature increases in the range of 10–100 K (Figure 5a). At temperatures above 100 K, this trend continues for the dilute stages but not for the dense stages, where instead a noticeable decrease in *T*_1e_ with an increase in temperature was observed (particularly for stage 1). The inverse line widths are plotted in Figure 5b, and they mirror the trends in the *T*_1e_ values with temperature; again, different trends are seen for dilute and dense stages.

What is surprising is that the line widths measured by cw EPR are sharper than those predicted on the basis of the measured *T*_1e_ values. For example, a *T*_1e_ of 66 ns (the value for stage 1 at room temperature) leads to an estimate for the line width of 1.07 mT, assuming that the *T*_2e_ is determined by the *T*_1e_. At 100 K, a *T*_1e_ of 125 ns corresponds to a calculated line width of 0.57 mT. The origin of these differences is discussed below.

### Magnetic Susceptibilities

The bulk magnetic susceptibilities for each stage were measured to help separate contributions to the EPR signals from metallic (Pauli)-like versus localized spins and to explore whether magnetic exchange interactions are responsible for some of the observed behavior. Stage 4 shows a diamagnetic response, while the other three are weak paramagnets. A turnover in the zero-field-cooled (ZFC) susceptibility at 15 and 25 K is seen for stages 1 and 2, respectively (see Figure S9). A Curie–Weiss fit of the plot of χ versus *T* for stage 1 (above 150 K, i.e., in the Curie–Weiss region) results in a Weiss constant θ of −88 K, suggesting that spin alignment starts around the turnover temperature seen in the *T*_1e_ data. Temperature-dependent (or Curie, *c*_C_) and independent components (Pauli, *c*_P_) were extracted from the fit: at 300 K, *c*_P_ = 8.4 × 10^–5^ emu K mol^–1^ and *c*_C_ = 3.6 × 10^–5^ emu K mol^–1^. Thus, Pauli paramagnetism dominates over Curie paramagnetism, as anticipated for a metallic system with negligible exchange interactions. The Curie contribution corresponds to 0.3 μ_B_ (Bohr magneton) per LiC_6_ unit.^53^

### HFEPR

HFEPR was used to investigate the local structure of lithiated graphite anodes. First, we note that all resonances observed here are due to spins on the lithiated graphite and not on any C additives. The resonances corresponding to pristine graphite or Super P Carbon could not be observed at this field. We tentatively attribute this to the very fast relaxation of the defect spins in these materials at such high fields.

**Figure 6:**
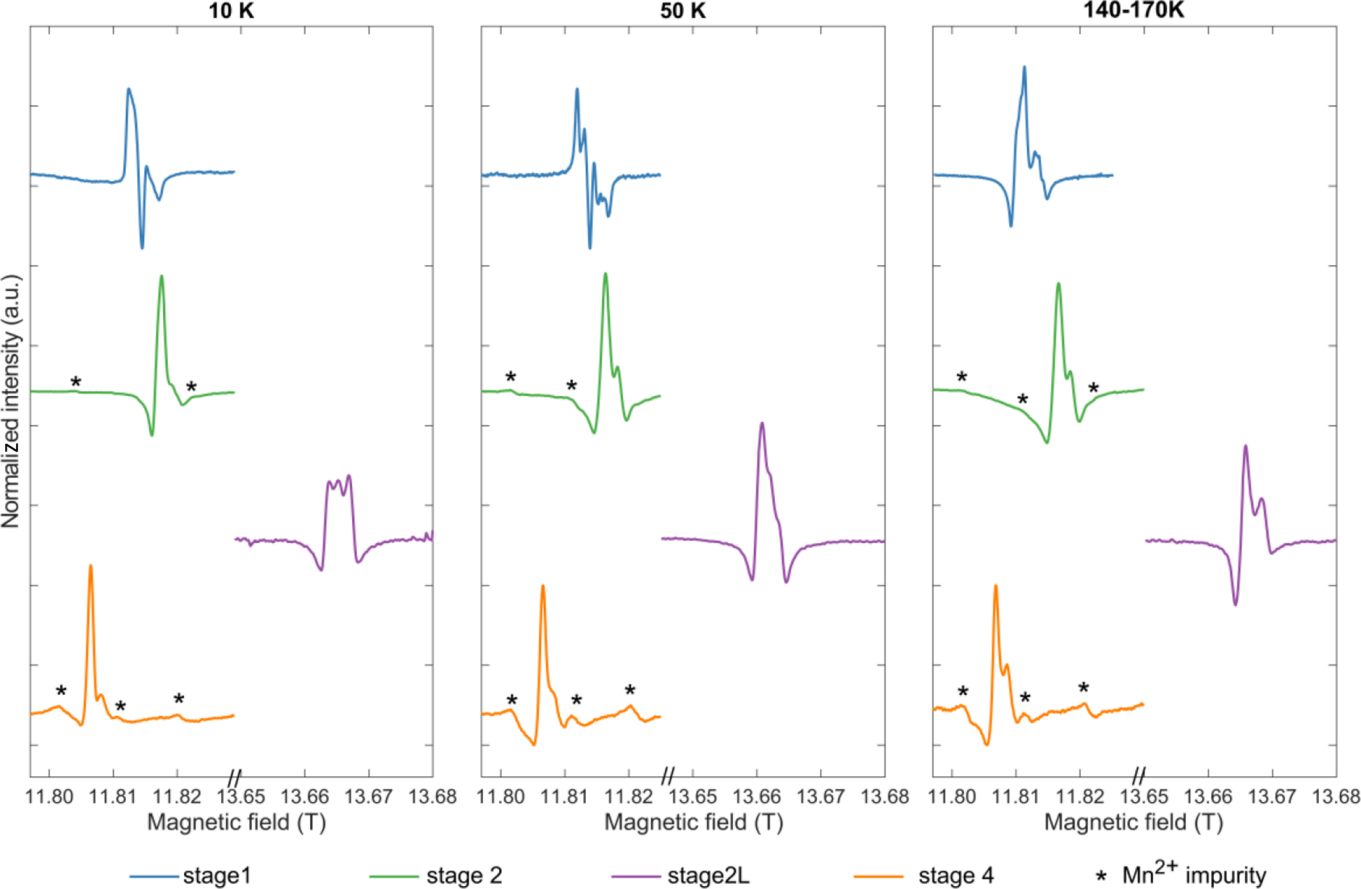
High-frequency EPR spectra of the four lithiation stages of graphite taken at 331 GHz (stages 1, 2, and 4) and 383 GHz (stage 2L; purple spectra). Three separate temperature points are presented to show the evolution of the signal with temperature. For the high-*T* spectra, stage 1 was collected at 158 K, stages 2 and 4 were collected at 140 K, and stage 2L was collected at 170 K (hence the range given). In the spectra of stages 2 and 4, hyperfine coupling from Mn^2+^ impurities in the capillaries can be observed and is indicated by asterisks.

**Figure 7:**
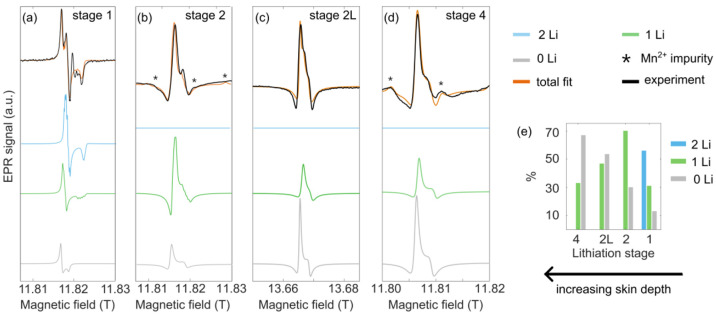
High-frequency EPR spectra of (a) stage 1, (b) stage 2, (c) stage 2L, and (d) stage 4 graphite taken at 331 GHz (stages 1, 2, 4) and 383 GHz (stage 2L) at 50 K. The spectrum for stage 1 was fitted to three different axial components, while the spectra of the remaining stages were fitted to two axial components, as outlined in the text. In the stage 2 and 4 spectra, peaks from Mn^2+^ impurities in the quartz capillaries are indicated by asterisks. (e) Weight percentage of the different components obtained from fitting HFEPR spectra for the four different lithiation stages at 50 K and 331 GHz (stages 1, 2, and 4) and 383 GHz (stage 2L).

HFEPR spectra were recorded for all samples at temperatures between 170 and 10 K (Figure 6) and frequencies between 331 and 441 GHz. The spectra recorded at 50 K showed the highest resolution across all samples, this being most pronounced for the stage 1 sample. This is consistent with the *T*_1e_ measurements obtained at lower fields where the stage 1 compound showed the longest *T*_1e_ at 50–100 K. Surprisingly, the spectra obtained from stages 1 and 2 could not be fit well unless Li hyperfine couplings were included in the simulations. The fits for the dense and dilute stages are presented in Figure 7, and the fitted **g** tensors, hyperfine couplings, and weights of the different components are listed in Table 1.

**Table 1:** Axial **g** Tensor Components for the Different Phases Deduced from Fitting the HFEPR Spectra of the Four Lithiation Stages at 331 GHz (stages 1, 2, and 4) and 383 GHz (stage 2L) at 50 K; Isotropic *g* Values Recorded at X-Band, Isotropic *g* Values as Reported by Lauginie and Co-workers, Hyperfine Coupling Constants Obtained from Fitting the HFEPR Spectra (in square brackets for the *x*, *y*, and *z* components)^a^

	two-Li component			one-Li component			zero-Li component					
stage	*g*_*x,y*_	*g_z_*	%	*g_x,y_*	*g*_*z*_	%	*g*_*x,y*_	*g*_*z*_	%	*g* (X-band, 100 K)	*g* (Lauginie et al.)^38^	*A* (MHz)
1	2.0043	2.0036	56	2.0044	2.0037	31	2.0045	2.0042	13	2.0133	2.0020	[5.0, 5.0, 4.4] (two Li), [0.2, 0.2, 13.5] (one Li)
2	–	–	–	2.0038	2.0033	70	2.0039	2.0034	30	2.0134	2.0022	[0.2, 0.2, 13.5]
2L	–	–	–	2.0043	2.0038	47	2.0045	2.0039	53	2.0138	2.0026	[2.5, 2.5, 1.0]
4	–	–	–	2.0054	2.0046	33	2.0054	2.0049	67	2.0145	–	[2.0, 2.0, 1.0]

a For a complete error analysis of fits to high-frequency spectra, see section 19 of the Supporting Information.

The 50 K spectrum at 331 GHz for stage 1 was fitted to three different axial systems, accounting for three distinct electron environments (Figure 7a); this model was verified by a fit to a spectrum at the same temperature but at a higher microwave frequency [441 GHz (Figure S13)]. The first environment (∼56% of the overall weight) with a *g*_iso_ of 2.0041 was modeled with isotropic hyperfine couplings of 4.8 MHz to two Li nuclei; we therefore attribute this resonance to electrons in the graphite layers with Li^+^ above and below the layer. The second resonance (∼31% of the total weight) at a *g*_iso_ of 2.0042 shows hyperfine coupling to only one Li nucleus (*A*_iso_ = 4.6 MHz), which we attribute to an electron on a graphite sheet with only one Li^+^ above or below it, i.e., a stage 2-like environment. Finally, the third axial environment (∼13% of the total weight) at a *g*_iso_ of 2.0044 shows no hyperfine coupling to Li. We therefore tentatively attribute this resonance to spins near defects: spins on or near the surface, perhaps from or near protonated and/or oxygenated C centers (C–H units, hydroxyls, and hydrogen carbonates), or trapped spins at C vacancies, or simply from electrons in graphitic sheets that are not near Li or where electron–nuclear self-decoupling has occurred (see below).

The spectra for stage 2 can be fitted to two environments, one with an electron coupling to one Li center and one that couples to no Li centers, with comparable *g* anisotropy and hyperfine splitting (see Table 1). The relative weights of the two components are ∼70% for the one-Li component and ∼30% for the zero-Li (defect) component. The line shapes appear to be phase-shifted in stage 2 compared to stage 1, which we ascribe to the greater conductivity of this phase at <200 K (Figure 4b); we show in the Supporting Information how by phase-shifting the stage 1 spectrum we can achieve similar line shapes (Figure S14). The spectra appear broader than in stage 1, which is likely a result of the shorter *T*_1e_ in stage 2 as measured by pulsed EPR (44% shorter than in stage 1 at 50 K).

In the dilute stages, only the one-Li component and zero-Li species are present, consistent with the expected layer stackings (Figure 7c,d). The relative concentrations of the three environments are compared in Figure 7e, with the zero-Li component becoming more dominant as the Li^+^ concentration decreases. We note that across the four stages these weights are not fully quantitative due to skin effects, but we believe they are sufficiently bulk sensitive to be meaningful [see the Supporting Information for further discussion of the effect of skin depth on the fitting (Figures S15 and S16)].

In our simulations, we included both Li isotopes (^6^Li and ^7^Li) at natural abundance. Additional spectra fitted exclusively with either ^6^Li or ^7^Li are shown in Figure S10a,b. Furthermore, we considered the possibility of there being hyperfine coupling to protons present as impurities in the graphite (e.g., at the edges of the graphene sheets) or possibly on the surfaces of the graphite in the solid–electrolyte interphase (SEI) formed upon electrolyte degradation (Figure S10c–e). See the Supporting Information for a full discussion. While we cannot rule out the possibility that a small subset of the unpaired electrons are coupled to protons, proton hyperfine coupling is not visible in the spectra of the dilute stages where these protons will also be present. Thus, while somewhat unexpected, we were unable to fit the high-field spectra of the stage 1 and 2 samples unless Li hyperfine couplings were included.

The fitted **g** tensors reproduce the trend seen in the X-band data across stages (Table 1), where a greater contribution of the Li band to the Fermi level results in **g** tensor components closer to *g*_e_. We note that the components of the **g** tensors are in general closer to *g*_e_ at high frequency than they are at X-band. This is tentatively ascribed to nonlinear Zeeman splitting at such high fields and to the decoupling of the SOC component from the electron spin (Paschen–Back effect).^54,55^

## Discussion

### Effect of Electrical Conductivity on EPR Parameters and Electronic Properties

The electronic properties of lithiated graphite depend on the nature of the electrons nearest the Fermi level, which are strongly influenced by local and long-range structure; these, in turn, dictate its electrochemical performance.

### Local Electronic Structure of Li-Intercalated Graphite

#### Contribution of the Li 2s Orbital to the Fermi Level

The *g* factors are probes of the density of states at the Fermi level in Li-intercalated graphite. As shown in Figure 4a, the **g** tensors at both X-band and high frequencies shift toward *g*_e_ from pristine graphite to fully lithiated (stage 1) graphite. As Li intercalates between the graphite sheets, electrons are introduced into the graphite conduction band and graphite is reduced, the contribution of Li 2s orbitals at the Fermi level increasing. The primary consequence of this is a reduction in the SOC component of the **g** tensors, resulting in the observed shift toward *g*_e_; this is also caused by the increased population of states further from the Fermi level of graphite, which are associated with values of *g*_∥_ closer to *g*_e_. This trend had been previously reported in the literature [albeit with slightly different*g* values (reported for the sake of convenience in Table 1)] by Lauginie and co-workers,^42^ who showed a similar decrease across the first three stages as the Li concentration increased.

#### Local Structure from HFEPR

While X-band EPR provided information about the bulk metallic properties of Li-intercalated graphite, HFEPR allowed us to investigate more closely the local electronic structure of these materials.

The high-frequency spectra (Figure 7a–d) can be fitted to three components (stage 1) and two components (stages 2, 2L, and 4), with two of these components (two Li and one Li) showing hyperfine coupling to Li (Table 1). We note that while hyperfine splittings are often more easily resolved at lower frequencies, where *g* anisotropy and *g* strain are less pronounced, they have occasionally been observed at a high frequency in, e.g., glass matrices.^56,57^ In our case, the higher fields allow the principal components of the **g** tensor, *g*_*x,y*_ and *g*_*z*_ to be resolved, in principle, making it easier to resolve the anisotropic hyperfine coupling constants. This is illustrated via simulations in the Supporting Information of the X-band spectra (Figure S12). The presence of three different environments in stage 1 is not surprising considering the difficulty in isolating pure stage 1 electrochemically.^51^ The isotropic hyperfine coupling constants for the two-Li (stage 1) components and one-Li (stage 2) component in the HFEPR fits are 4.8 MHz and between 3 and 5 MHz, respectively (for a full error analysis, see section 19 of the Supporting Information). The larger principal component of the hyperfine tensor in the 1 Li environment (*A*_*z*_ = 13.5 MHz) is assigned, at least in part, to a change in the interlayer spacing (it increases from stage 2 to stage 1).^58^ This difference results in a change in the overlap of Li^+^ orbitals and carbon-based orbitals near the Fermi level. Additionally, we note that in a stage 2-type Li environment, the electron is shared between the carbon layers above and below; in a stage 1 compound, while this is still the case, the carbon layers also are bound to one Li above and below the carbon layers, i.e., the electron is shared over two Li ions. Thus, intuitively, the contribution of the Li 2s orbital to primarily the 2p carbon band will be larger.

These hyperfine coupling constants can be compared with the ^7^Li NMR Knight shifts (see Figure S2), where stage 2 has a larger shift (45.0 ppm) compared to that of stage 1 (42.6 ppm), also suggesting a greater population of the Li 2s orbitals at the Fermi level for stage 2.^13^ The NMR Knight shift (δ_KS_) can be correlated to the isotropic hyperfine coupling constant, *A*_iso_, via the following equation:^28,59^
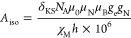
6where *N*_A_ is Avogadro’s constant, μ_0_ is the vacuum permeability, μ_N_ is the nuclear magneton, *g*_N_ is the nuclear *g* factor, χ_M_ is the molar susceptibility (cf. Figure S9) at 50 K (the temperature of the HFEPR spectrum), and *h* is Planck’s constant. Using eq 6 and the experimental NMR shifts, we calculated an *A*_iso_ of 4.6 MHz for stage 1 and an *A*_iso_ of 3.4 MHz for stage 2. While further EPR experiments with, for example, samples containing only ^6^Li or with different concentrations of defects will help us test alternative hypotheses, it is remarkable that the fitted EPR spectra and NMR Knight shifts and measured susceptibilities result in hyperfine constants that are on the same order of magnitude.

These hyperfine coupling constants should be contrasted with the much larger isotropic hyperfine coupling constant of 52.7 MHz for Li metal, calculated here using the ^6,7^Li Knight shift, δ_KS_, of 245 ppm and a susceptibility, χ_M_, of 1.99 × 10^–10^ m^3^ mol^–1^.^60^ Hence, the hyperfine coupling constants for lithiated graphite confirm that the Li ions are partially charged and not present as Li^0^, consistent with predictions made based on the NMR (Knight) shifts. If the additional electrons inserted into the conduction band associated with the Li intercalation had all been located on the Li ions, much larger hyperfine couplings would be expected.

Finally, we note that the hyperfine coupling constant extracted by EPR reflects an admixture of both Fermi-contact and dipolar components due to electron–nuclear dipolar interactions not being averaged, e.g., by spinning in NMR, accounting for the large anisotropy in *A*. On the contrary, the hyperfine shift obtained through MAS NMR is solely due to the Fermi-contact shift, as the dipolar interaction is averaged out.

Finally, we should consider the question of why hyperfine coupling constants are seen despite the very rapid mobility of electrons, which diffuse over very long distances on the time scale of the hyperfine interaction.^32^ We note here that we see both Curie and Pauli contributions to the magnetism (i.e., a temperature-dependent term and a temperature-independent term). One possibility is that in these disordered graphites a subset of the electrons in the carbon sheets are similarly partially localized near defects, contributing to the Curie component and resulting in observable Li hyperfine coupling constants if the Li ions are near these defects. However, it should be noted that when hard carbons, which contain highly disordered graphene sheets, are lithiated (or sodiated), sites near the defects are lithiated (sodiated) first, i.e., at higher voltages,^61^ which means we might expect to see this phenomenon at higher states of charge, not just in the stage 1 and 2 compound. A study of single graphene sheets has discussed the role that defects [nonbonding orbitals (NBOs)] play in controlling EPR spectra and magnetic susceptibility proposing that the role of NBOs will depend strongly on the degree of hole or electron doping in the graphene sheets.^62^ Future high-field EPR studies will focus on different graphites with differing degrees of disorder and particle sizes and potentially with different degrees of isotopic enrichment.

#### Metallicity

X-Band EPR spectra can provide (semi)quantitative information about the metallicity of lithiated graphite through the *A*/*B* ratios and electron relaxation times.

The electrical conductivity of the graphite intercalation materials is affected by the following factors: carrier concentration, electron hopping between defects states (from defects or dopants), which is thermally activated, and the presence of phonons, both of which reduce the electron mean free path, scattering the electrons and increasing the resistivity.^5^ Unlike pristine graphite, where temperature plays a substantial role in carrier generation, lithiated graphite is expected to have a higher temperature-independent carrier concentration due to the higher density of states at the Fermi level within the conduction band. Graphite’s layered, anisotropic structure results in different in-plane and out-of-plane conductivities, with the *a*/*c* conductivity ratio decreasing substantially upon lithiation, as discussed above.

In EPR, the metallic character of lithiated graphite and therefore the presence of skin effects result in an *A*/*B* of >1. The *A*/*B* parameter depends on the conductivity and the EPR frequency (eq 3), less conductive samples and/or low microwave frequencies increasing the skin depth and therefore the number of electrons probed by EPR. *A*/*B* also depends on the size of particles and their packing density, as this also affects the ability of the microwaves to penetrate into the sample.^32^ This phenomenon was clearly observed for stage 1 packed loosely in a capillary versus a rotor (Figure S8), where *A*/*B* ratios of 1.96 and 3.80, respectively, were seen. Because all of the samples were made from the same pristine graphite sample and assuming similar packing, the *A*/*B* ratio is a probe of the relative conductivity between samples and its temperature dependence. As shown in Figure 4b, the *A*/*B* ratio of the dense stages (1 and 2) is larger than that of the dilute stages (2L and 4) across all temperatures. The *A*/*B* ratio increases as the temperature is decreased for all stages, indicating increasing conductivity. However, the rate of change in the *A*/*B* ratio is smaller for stage 1, indicating that it is a better metal. In intercalation compounds involving heavier alkali metals, the change in resistivity, ρ, has been fit with a function of the form ρ = *a* + *bT* + *cT*^2^, the terms linear in *T* and *T*^2^ being ascribed to phonon–electron and electron–electron scattering processes, respectively, both processes becoming more frequent as the temperature is increased.^5,6^ Given that the *A*/*B* ratio depends on *d*/δ, and that δ is proportional to σ^–1/2^ (eq 3), and thus ρ^1/2^, a strong dependence of the *A*/*B* ratio on temperature might similarly be expected. Attempts to fit the *A*/*B* ratio using a temperature dependence of a form used to describe ρ^–1/2^ were not, however, successful, the *A*/*B* ratio becoming less (rather than more) dependent on temperature closer to room temperature. This is shown in the Supporting Information, where the *A*/*B* ratio was fitted to two close-to-linear regimes, above 200 K and below 200 K for stage 2 and approximately 210 K for stages 2L and 4 (Figure S17 and Table S4), to illustrate the weakened temperature dependence at higher temperatures. This phenomenon may be simply due to the fact that above ∼210 K skin depth δ starts to approach the sample thickness, *d*, the regime where the *A*/*B* ratio is less sensitive to any changes in δ.

It should be noted that the skin depth was estimated above without taking into account any anisotropy in the conductivity. While there is some scatter in the data, likely because of the difficulty in synthesizing fully lithiated (pure-phase) LiC_6_, conductivities in the *a −b* plane of 2.4 × 10^5^ S cm^–1^ at room temperature have been measured (increasing by a factor of ∼5 at 100 K), while the conductivity at room temperature in the perpendicular (*c*) direction is ∼14 times lower.^6,63^ If we consider that the surface areas of most polycrystalline graphites [including that used here (see Figure S1)] are dominated by the *a–b* basal planes, then the skin depth perpendicular to the *a–b* planes is actually 4 μm at room temperature (cf. 1 μm parallel to the planes) and indeed on the order of magnitude of many of the particles’ radii; thus, X-band microwaves can penetrate through the graphite basal planes deep into the bulk of the samples at room temperature. However, a factor of 5 increase in basal plane conductivities has been observed with a decrease in temperature to 100 K in stage 1 and 2 Li compounds, and thus, skin depths will be more pronounced at lower temperatures. Increased overlap between the carbon layers (associated with the contraction seen in the *c*-direction, particularly in the lower stages) should similarly be associated with a higher conductivity.

The measured values for the *A*/*B* ratio close to 2 are consistent with *d* and δ values on the same order of magnitude (independent of the theory and assumptions about sample geometry used to derive expressions for *A*/*B* vs *d*/δ).^32^ Using a skin depth of 4 μm and an average particle diameter of 13 μm produces a *d*/δ ratio of ∼3.25 for stage 1 (at room temperature) and (from Figure 15 of ref (32)) an estimated *A*/*B* ratio of ∼2, consistent with the value measured here.

#### Electron Relaxation Times

The effect of metallicity is also observed in the electron relaxation times. Two main phenomena affect *T*_1e_ and *T*_2e_: skin effects and collisions with defects. Both phenomena are influenced by the electron mobility.

The *T*_1e_ values (measured by pulsed EPR) track the *T*_2e_ values (determined from the X-band line width) but are approximately 2–3 times shorter [the effect is more pronounced for stages 1 and 2 than for stage 2L or 4 (see Table S5)]. This likely reflects how the *T*_1e_ is measured. Either a saturation or 180° pulse is first applied, during which the electrons move in and out of the skin depth region, feeling the effect of the pulse at random intervals and with varying power levels.

One relevant parameter is *T*_D_, the time to move out of the skin depth region. If we assume that one-dimensional diffusion in the *c*-direction is the most relevant, then using the values for LiC_6_ of the electron diffusivity, *D*, in the *c*-direction of 18 cm^2^ s^–1^ (from ref (42)) and the skin depth in the *c*-direction [δ = 4 μm (from the *c*-conductivity at RT)] then from eq 5*T*_D_ in the *c*-direction is ∼2 ns. This is much shorter than the 16 ns 180° pulse used in the inversion recovery experiment, and a tip angle of 180° will not be achieved, particularly for large particles. Furthermore, the resonances of all stages are also too broad for them to be effectively excited. Thus, the induced magnetization will take less time to return to equilibrium following the pulse, and the measured *T*_1e_ is shorter than the true value. Similarly, a long saturation pulse will likely not completely saturate the signal again because the resonance is too broad and because of skin depth issues (some residual magnetization remains, and a shorter time is required to reach equilibrium). Thus, it is perhaps more appropriate to refer to these measured *T*_1e_ values as effective or scaled *T*_1e_ values (or *T*_1e_*). Despite the challenges in measuring the *T*_1e_, *T*_2e_, as measured from the line width, will contain contributions from the *g* anisotropy; hence, it is still useful to consider the *T*_1e_* values.

The *T*_1e_ (and *T*_2e_) values are affected by mean free path *a* before a collision with defects and also the probability that a collision will lead to a change of spin, together quantified via the spin depth. There are two types of defects, extrinsic dopants/defects and those caused by interactions with lattice phonon modes. In general, the SOC decreases with an increase in lithium content, the SOC helping to drive spin flips. Consistent with this, Lauginie et al. observed that the *T*_2e_ values (as determined from the line width) of stage 1 and stage 2 graphite compounds decreased with an increase in the atomic number of the intercalant atom (from Li to Rb), consistent with the SOC mechanism.^42^ This mechanism is also consistent with our generally longer *T*_1e_* (and *T*_2e_) values for stage 1 and with its small *g* values (which again indicate a smaller SOC). Elliot showed that *T*_2e_ was proportional to *T*_R_/(*Δg*)^2^, where *T*_R_ represents the time between collisions (a function of the electron diffusivity and mean free path *a*), and *Δg* is the deviation from *g*_e_. The shorter *T*_1e_ values of the higher stages correlate to a degree to the greater carbon 2p_*z*_ contribution to the density of states at the Fermi level.

With a decrease in temperature, the time between collisions with phonons increases, and we would expect the *T*_1e_* to increase. This trend is observed for stages 1 and 2 (down to 100 K) but not for the less dense stages. Instead, for stages 2L and 4, *T*_1e_* gradually decreases with temperature. The two different trends for the *T*_1e_ values of the dilute and dense stages suggest that metallicity plays a more significant role in the electron relaxation of stages 1 and 2. Furthermore, stages 1 and 2 have greater three-dimensional conductivity due to Li^+^ sitting between the graphite layers.

The *T*_1e_ values decrease below approximately 100 K in stages 1 and 2. This turnover in the dense stages correlates with the magnetometry data, where antiferromagnetic ordering at 15 and 25 K was seen for stages 1 and 2, respectively (see Figure S9). The magnetic response can be divided into a Curie and temperature-independent (Pauli) component; on cooling, the electron spin fluctuations will diminish as the thermal energy decreases, and eventually their magnitude is no longer sufficient to overcome the local exchange constants, likely resulting in the development of (some) locally ordered spin clusters, reducing the Curie component of the susceptibility. Rapid motion of the electrons results in collisions with these localized spins/clusters and an additional mechanism for reducing *T*_1e_, when the local fluctuations of these clusters enter the *T*_1e_ time scale (the X-band Larmor frequency).^42,43,64^ As a result, the relaxation times initially increase with a decrease in temperature (due to fewer phonon scattering effects) and then decrease at low temperatures due to magnetic exchange effects; a maximum in *T*_1e_ is therefore seen for these samples.

In contrast, no turnover in *T*_1e_ is seen for the dilute stages, suggesting an absence of (significant) exchange coupling effects, consistent with the lower electron spin concentration at these lower lithiation levels; this is consistent with their much lower ZFC susceptibilities (Figure S9).

#### Local Structure and Skin Effects

**Figure 8:**
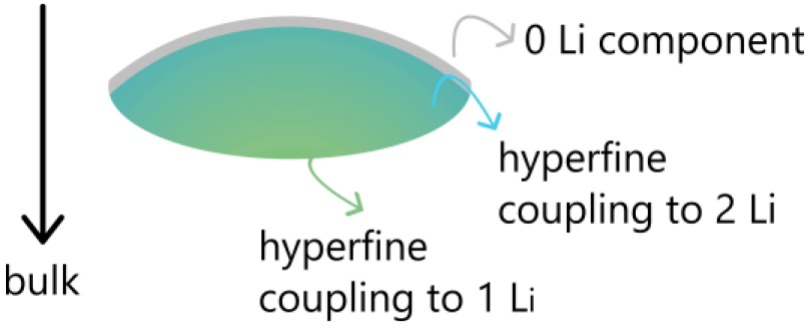
Visualization of the Li concentration gradient in a stage 1 particle based on the weights of the different components of the fit as reported in Figure 7e.

An important consequence of the metallicity of charged graphite is the presence of skin effects, which determine the relative weights of the different environments that are observed especially at higher frequencies and low temperatures, where the skin depth is significantly reduced (eq 3, where δ is ∼0.5 and 0.7 μm at 331 GHz and 300 K for the skin depth in the *ab*- and *c*-directions, respectively). Analysis of the effect of skin depth on HFEPR spectra taken at 10, 15, and 50 K (Figures S15 and S16) provides a spatial map of the different components, with the defect spins being at or closer to the surface (because the weight of this component remained constant across the three temperature points examined here, the skin depth decreasing as the temperature is decreased and conductivity increases), followed by the two-Li component, and with the one-Li component toward the core. This suggests that lithiation is associated with a concentration gradient, with the core being less lithiated than the shell of the particle (Figure 8). Thus, HFEPR probes the heterogeneity in lithiation, the results indicating that it is difficult to fully lithiate the center of the particle. The presence of the one- and two-Li environments in the stage 1 sample, the one-Li environment having a *g* factor different from that seen for the stage 2 material, suggests that the one-Li environment is different in the two materials and affected by the presence of two-Li environments. This suggests that at least some of these one-Li environments in the stage 1 compound are present as stacking faults and thus are in the proximity of the two-Li environments.

## Conclusions

In this work, we aimed to improve our understanding of the electronic structure of Li-intercalated graphite at different stages, namely, dense stages 1 and 2 and dilute stages 2L and 4, by using a variety of EPR methods. Electrochemical cycling and ^7^Li NMR were also used to assess phase purity and to contextualize the results in the battery domain.

Variable-temperature X-band EPR was sensitive to the degree of metallicity of the four different stages, with the temperature dependence of the *A*/*B* ratios indicating a greater electrical conductivity and more significant skin effects at low temperatures across all stages. The dense stages showed more metallic behavior than the dilute stages, the *A*/*B* ratio increasing with a decrease in temperature, consistent with higher conductivities. The metallic character introduced by Li intercalation is also reflected in the observed *g* factors, where the greater contribution of the Li 2s band to the density of states at the Fermi level is responsible for the shift in the measured *g* factors toward *g*_e_ on lithiation.

X-Band pulsed EPR was used to estimate the longitudinal electron relaxation times, *T*_1e_, which show different temperature dependence for the dilute and dense stages and which were explained in terms of the effect of phonons and defects on scattering processes and the magnetic properties of the samples.

The measured *T*_1e_ values are also fundamental in assessing the optimal conditions for dynamic nuclear polarization (DNP) NMR experiments, where the electron polarization is transferred to nearby nuclei, enhancing their NMR signal. The longest *T*_1e_ values are seen for stage 1 compounds at ∼100 K.

Finally, HFEPR showed the presence of hyperfine coupling between the metallic electrons and Li observed here for the first time, with values indicating that the electrons in lithiated graphite largely occupy carbon-based orbitals. HFEPR showed that the electrochemically isolated stage 1 sample also contained stage 2-like local environments, indicating the presence of graphitic interlayers containing no Li. The relative weight of these two components changed as a function of temperature due to changes in conductivity and thus skin depth effects, indicating that more stage 2 environments are present in the bulk of the particles, reflecting the difficulty in fully lithiating graphite. Further studies are, however, required to understand the role that delocalized (metallic) and more localized spins/clusters play in giving rise to the electron–lithium hyperfine couplings observed at high fields.

Our work demonstrates the power of EPR spectroscopy in investigating the local electronic structure of graphite on cycling, paving the way for this technique to be used as a tool for investigating the electronic properties of novel materials for use in lithium-ion batteries. This work also sets out a framework for screening materials for DNP, a tool for selective excitation of species near surfaces (for example the solid–electrolyte interphase)^65,66^ and/or paramagnetic centers (for example, in the bulk of cathodes and solid electrolytes).^67,68^

## Supporting Information Available

Solid-state NMR spectra, additional high-frequency parameters and data, magnetometry data, and additional fits to the X-band and high-frequency spectra (PDF)
