# Supplementary material for: Graphite Anodes for Li-Ion Batteries: An Electron Paramagnetic Resonance Investigation
Source: Chem Mater. 2023 Jul 13;35(14):5497–511. doi: 10.1021/acs.chemmater.3c00860 (PMC10373490; doi:10.1021/acs.chemmater.3c00860)
Supplement: Supplementary file 1 — cm3c00860_si_001.pdf [file cm3c00860_si_001.pdf]

Supplementary information

Graphite anodes for Li-ion batteries – an electron paramagnetic resonance investigation

Teresa Insinna,[1] Euan N. Bassey,[1,2] Katharina Märker,[1,3] Alberto Collauto,[4] Anne-Laure Barra,[5,6] Clare P. Grey[1,*]

[1] Yusuf Hamied Department of Chemistry, University of Cambridge, Lensfield Road, Cambridge, CB2 1EW, United Kingdom

[2] Current address: Materials Research Laboratory, University of California, Santa Barbara, Santa Barbara, CA 93106-5121, United States

[3] Current address: Univ. Grenoble Alpes, CEA, IRIG, MEM, Grenoble 38000, France

[4]Centre for Pulse EPR (PEPR), Imperial College London, London W12 0BZ, United Kingdom

[5]LNCMI-CNRS, EMFL, Univ. Grenoble-Alpes, 25 Rue des Martyrs, B.P. 166, 38042 Grenoble Cedex 9, France

[6]LNCMI-CNRS, EMFL, Univ. Toulouse 3, Insa Toulouse, 118 Route de Narbonne 31062 Toulouse Cedex 9, France

Contents

Supplementary information 1

1 Scanning Electron Microscopy of Hitachi Graphite particles 3

2 Solid state NMR spectra of the 4 stages 4

3 Experimental details of the HFEPR spectra 5

4 *g*-factor calibration for HFEPR 6

5 Phase cycling used for Pulsed EPR 7

6 Fitted cw spectra of pristine graphite 8

7 EPR signal of other electrode components 9

8 Example of VT EPR spectra 10

9 Effect of sample packing on *A/B* ratios 11

10 Magnetometry measurements 12

11 Additional fits for stage 1, 331 GHz, 50 K, accounting for ^{7}Li, ^{6}Li, ^{1}H hyperfine coupling.. 13

12 Simulation at X-band with high-frequency parameters 15

13 Fit of the dilute stages 15

14 Fit at 441GHz of stage 1 sample 16

15 Effect of phase on HF fitting 17

16 Fit of Stage 1 at 10 K and 25 K 18

17 Fits of two regimes of electrical conductivity through *A/B* ratios 20

18 T_{2e}s derived from the X-band linewidths 21
19 Error in fitted Hyperfine coupling constants for stage 1 at 331 GHz 22
References 23

1 Scanning Electron Microscopy of Hitachi Graphite particles

Scanning electron microscopy (SEM) images were taken of the Hitachi MagE3 to assess particle morphology and measure particle size. They were taken on a Tescan MIRA3 FEG-SEM microscope. An example is provided in Figure S1. The average particle size was measured as 13 μm by averaging over 100 particles using ImageJ software.

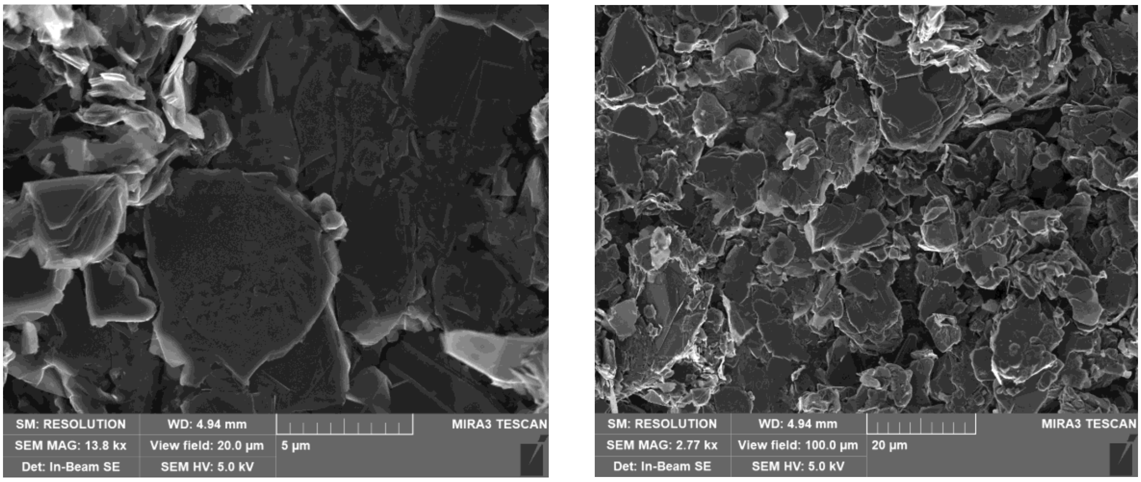

Figure S1. SEM images of Hitachi MagE3 graphite particles. While there is considerable distribution in sizes the average particle size was found to be 13 μm.

2 Solid state NMR spectra of the 4 stages

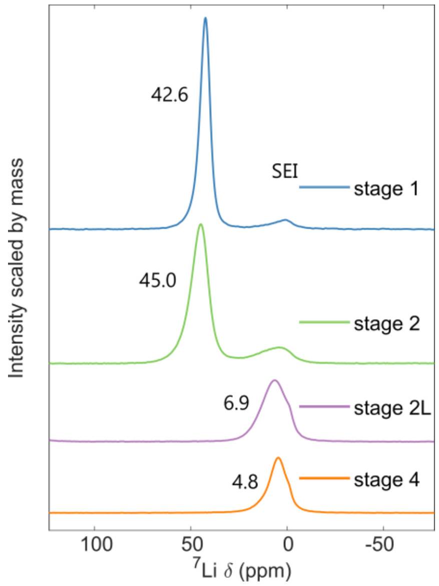

Figure S2. ^{7}Li solid state MAS NMR spectra of the four stages examined in this work.

Solid state ^{7}Li NMR spectra were measured for the four stages to assess sample purity as described in the Experimental. The observed shifts are consistent with stages 1, 2, 2L and 4 as reported e.g. by Letellier and co-workers.[1]

3 Experimental details of the HFEPR spectra

The experimental parameters used to collect the 331 GHz spectra (stages 1, 2, 4) and 383 GHz (stage 3) at the temperatures shown in the main text are reported in the Table S1 below.

Table S1. HFEPR experimental parameters for the high frequency spectra reported in the main text.

Sample	Modulation field (Hz)	Modulation Amplitude (mT)	Scan rate (mT s^{-1})
Stage 1	643	0.42	0.2
Stage 2	687	0.84	0.3
Sage 3	643	0.84	0.5
Stage 4	687	0.84	0.3

4 *g*-factor calibration for HFEPR

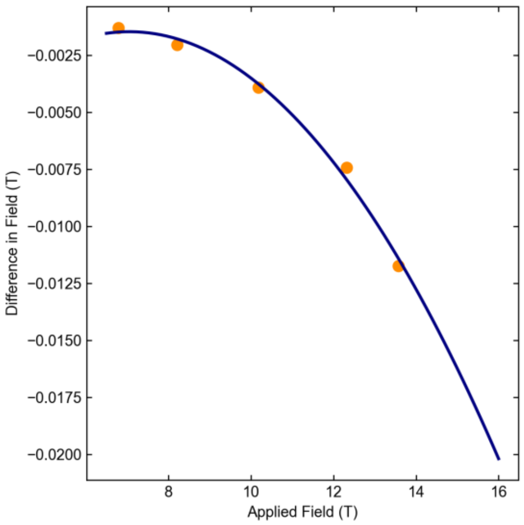

Figure S3. Calibration curve obtained from the signal of a Mn(II) in MgO sample at different field and related polynomial fit.

The high frequency *g*-factors were corrected using a field calibration factor obtained by measuring the difference in the resonance field between the measured and the expected field for Mn(II) in MgO.[2] This was done over a range of fields and the difference in field was plotted against the resonance field (Figure S3). The curve was then fitted to a quadratic polynomial and the coefficients *a*, *b*, *c* were then substituted in the following expression for the "corrected" field

$$B_{out} = B_{in} + (aB_{in}{}^{2} + bB_{in} + c).$$

The "corrected" *g*-factor was then obtained from B_{out}.

5 Phase cycling used for Pulsed EPR

Here we report the phase cycling used to collect FID and Inversion/saturation recovery spectra

Table S2. Phase cycling for FID and Inversion recovery and saturation recovery spectra

FID

Step	φ(π/2)	Detection phase
1	+x	+1 (+a/+b)
2	+y	+i (+b/-a)
3	-x	-1 (-a/-b)
4	-y	-i (-b/+a)

Inversion/saturation recovery

Step	φ(π)	φ(π/2)	Detection phase
1	+x	+x	+1 (+a/+b)
2	+x	+y	+i (+b/-a)
3	+x	-x	-1 (-a/-b)
4	+x	-y	-i (-b/+a)
5	+y	+x	+1 (+a/+b)
6	+y	+y	+i (+b/-a)
7	+y	-x	-1 (-a/-b)
8	+y	-y	-i (-b/+a)
9	-x	+x	+1 (+a/+b)
10	-x	+y	+i (+b/-a)
11	-x	-x	-1 (-a/-b)
12	-x	-y	-i (-b/+a)
13	-y	+x	+1 (+a/+b)
14	-y	+y	+i (+b/-a)
15	-y	-x	-1 (-a/-b)
16	-y	-y	-i (-b/+a)

6 Fitted cw spectra of pristine graphite

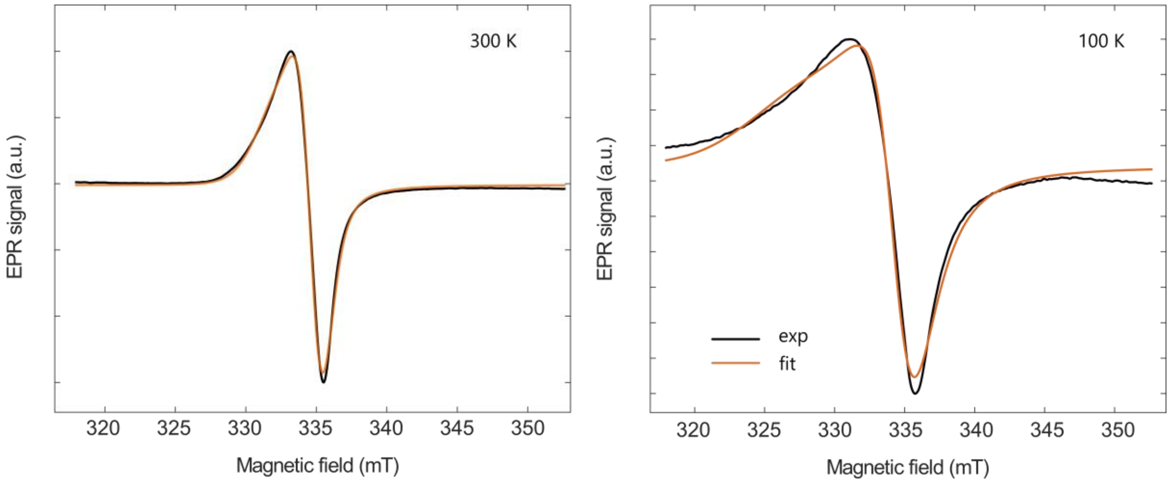

Figure S4. Experimental and fitted spectra of pristine graphite recorded at X-band at 300 K (left) and 100 K (right). The fit was performed using the Easyspin toolkit for Matlab.

The variable temperature EPR spectra of graphite are reported in Figure 3 in the main text. Here we propose the fits performed using EasySpin software on the 300 K and 100 K spectra (Figure S4). *g*-factor, *g*-anisotropy and peak-to-peak linewidth increase with lower temperature as reported previously in the literature.[3,4] The fit parameters are reported in the table below (Table S3).

Table S3. *g*-factors, linewidth and phase from fit EPR lineshapes of pristine graphite at 300 K and 100 K

Fit parameter/ Temperature	$g_{\perp}$	$g_{\parallel}$	Linewidth (peak-to-peak) / mT	Phase / rad
300 K	2.0228	2.0406	0.82	0.13
100 K	2.0253	2.0741	2.39	0.16

7 EPR signal of other electrode components

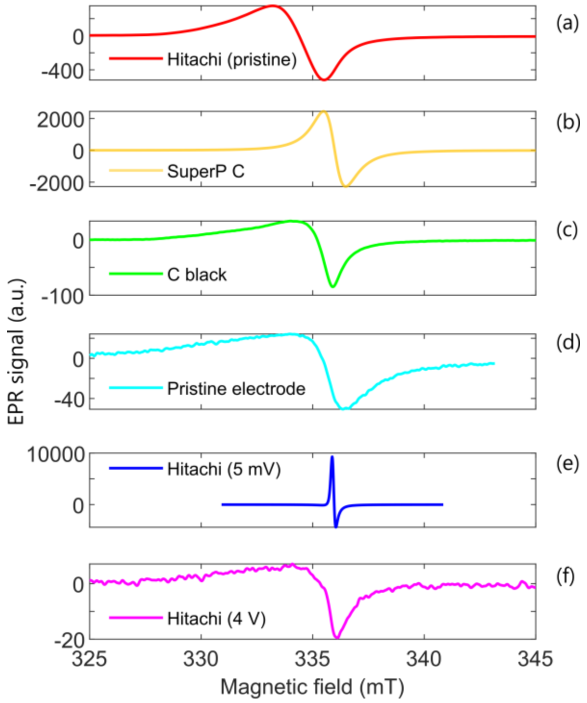

Figure S5. Room temperature EPR spectra of (a) pristine Hitachi MagE3 graphite, (b) Super Porous C, (c) C black, (d) a pristine Hitachi graphite electrode with the formulation described in the experimental section, (e) an electrode charged to stage 1, (f) an electrode oxidised to 4 V. The spectra intensities are normalised by mass.

The conductive carbons often used to cast battery electrodes also have an EPR signature (Figure S5). The spectra have been normalized by mass. It can be seen that the absolute intensities of the conductive carbon additives are several orders of magnitude lower than the intensity of lithiated graphite (e). The signal shifts to higher field as Li is intercalated, consistent with a smaller *g*-shift as expected from contribution of the Li band to the Fermi level, and its intensity is 2 order of magnitudes larger than in pristine graphite.

8 Example of VT EPR spectra

An example of a variable temperature series is illustrated below in Figure S6.

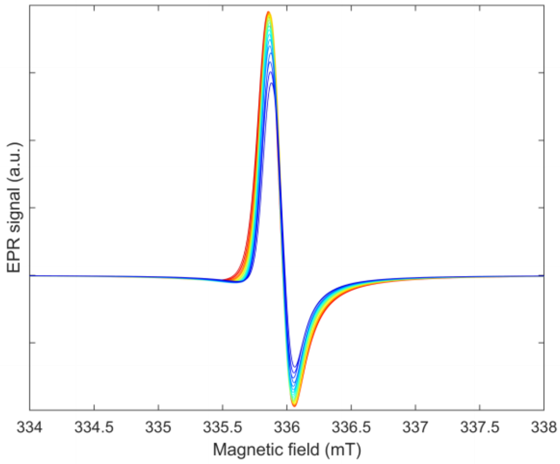

Figure S6. VT EPR spectra of stage 2 graphite collected at X-band between 300 K (red) and 100 K (blue).

The spectra at 100 K of the four stages studied here are shown in Figure S6 below.

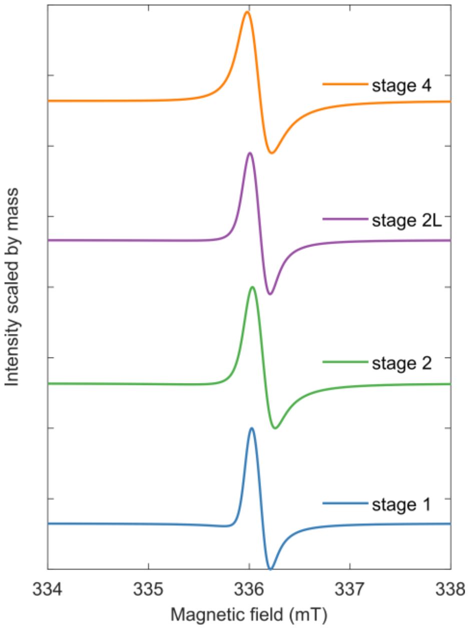

Figure S7. X-band spectra of the four stages collected at 100 K.

9 Effect of sample packing on *A/B* ratios

The effect of sample packing on the asymmetry parameter was explored by comparing spectra of LiC6 as a loosely packed powder (standard method used in the main text) and as tightly packed in a rotor (Figure S8). The densely packed sample showed an *A/B* ratio of 3.80, while the loosely packed sample had *A/B* = 1.96.

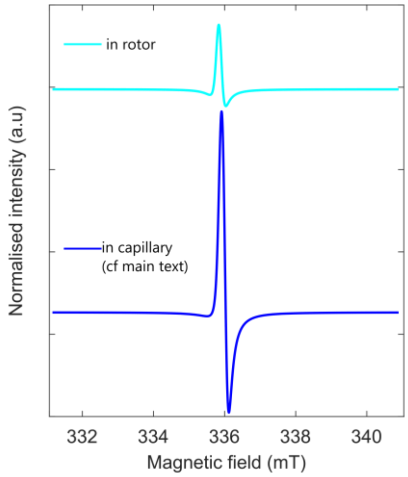

Figure S8. EPR spectra of LiC_6 measured at 300 K in a rotor (tightly packed powder) and in a standard EPR capillary (loosely packed).

10 Magnetometry measurements

Bulk magnetic susceptibility measurements on lithiated graphite have been previously carried out by Mukai and co-workers.[5] However, variations in structural compositions can lead to different magnetic properties which is why these measurements were also carried out here.

The zero-field cooled magnetometry profiles of the four stages examined in this work were acquired on powder samples from cycled anodes (approximately 10 mg) using a Quantum Design Magnetic Property Measurement System 3 (MPMS) superconducting quantum interference device (SQUID) magnetometer. The zero-field cooled (ZFC) susceptibilities were measured in a field of 0.1 T over a temperature range 2–300 K. As $M(H)$ is linear in this field range, the small-field approximation to the susceptibility, $\chi \simeq M/H$, was assumed to be valid.

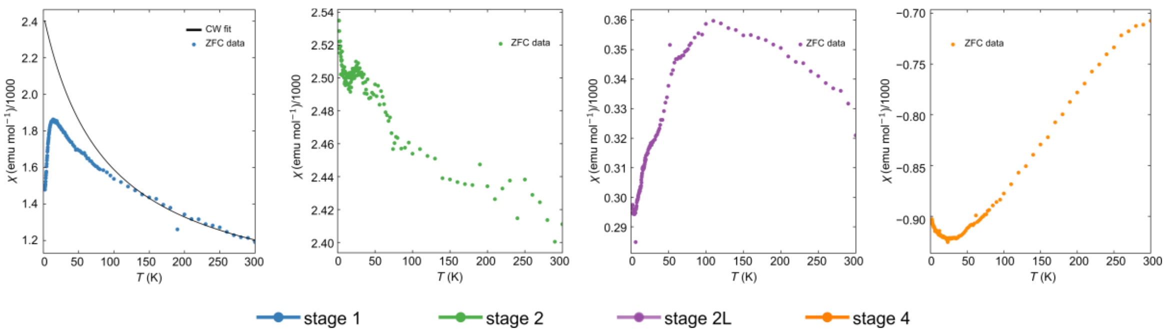

Figure S9. Zero-field cooled magnetometry of the four lithiation stages examined in this work. Stages 1 and 2 show antiferromagnetic ordering at 15 K and 25 K. For stage 1 a Curie-Weiss fit was attempted in the paramagnetic region, producing a Weiss constant of -88 K.

The ZFC profiles are showed in Figure S9. Stages 1 and 2 show antiferromagnetic ordering at 15 K and 25 K respectively. Stage 2L shows possibly 2D ordering at 110 K, while stage 4 appears essentially diamagnetic.

11 Additional fits for stage 1, 331 GHz, 50 K, accounting for ^{7}Li, ^{6}Li, ^{1}H hyperfine coupling

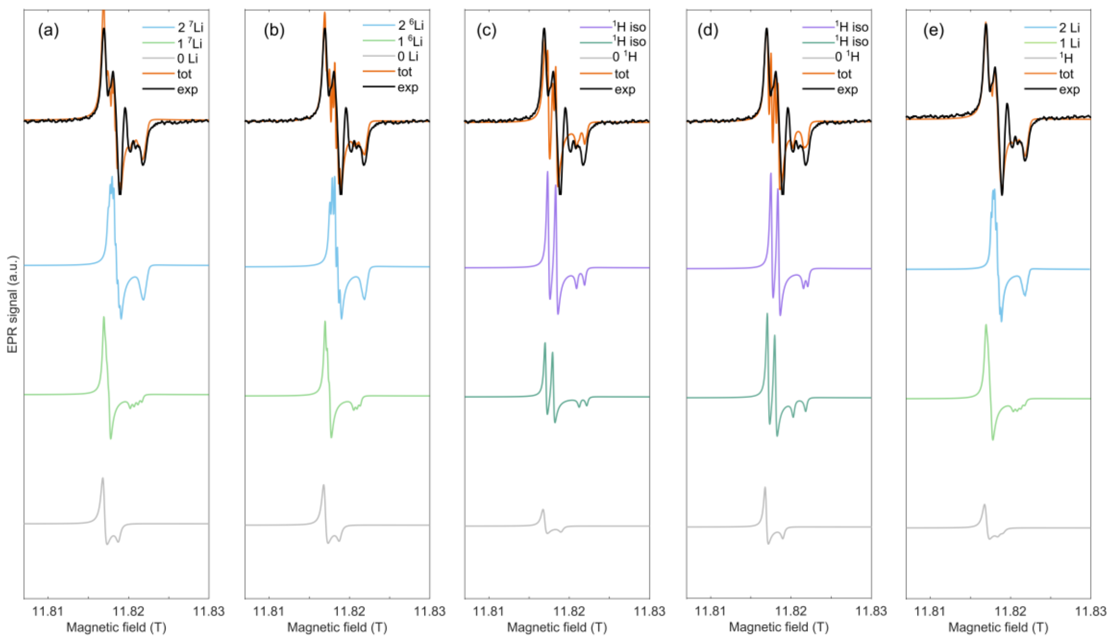

Figure S10. Stage 1 spectrum taken at 331 GHz at 50 K with simulated three axial environments as follows: (a) 2 Li and 1 Li environments only considering ^{7}Li isotope; (b) 2 Li and 1 Li environments only considering ^{6}Li isotope; (c) 2 Li and 1 Li components replaced by isotropic H hyperfine coupling; (d) 2 Li and 1 Li components replaced by axial H hyperfine coupling; (e) 2 Li and 1 Li components as in main text (hyperfine to Li at natural abundance) and 0 Li component showing axial H hyperfine coupling.

Additional fits to the 331 GHz sage 1 spectrum recorded at 50 K were performed to assess the effect of different Li isotopes (^{6}Li and ^{7}Li) and to explore the presence of hyperfine coupling to protons in the solid electrolyte interphase (SEI) formed during cycling and/or to potential C-H groups in graphite. The fits to ^{6,7}Li show how the ^{7}Li component dominates the spectrum in the main text (as expected from the larger natural abundance, 92.58%, of ^{7}Li) with the ^{6}Li aiding the fitting of the higher field part of the spectrum in particular (Figure S10 (a,b)).

Fits which include hyperfine coupling to H instead of Li are shown in Figure S10 (c, d), as isotropic and axial hyperfine coupling respectively. This represents the scenario of proton impurities dominating the EPR spectrum and the unpaired electron spins being present at C-H sites. The fits do not fully match the hyperfine splittings seen in the higher field portion of the spectrum, indicating this model is not representative of the full electronic structure of stage 1 graphite. The fitted hyperfine coupling constants range between 20-30 MHz. Such result is unsurprising as a battery-grade graphite was used in this work, and such graphites are expected to have a minimal number of functional groups (C-H units, hydroxyls and hydrogencarbonates) to be minimal compared to e.g. activated carbons and hard carbons, as in the graphitisation process most impurities are burned off.

The last fit (Figure S10 (e)) shows the scenario where the 2 Li and 1 Li environments (at natural abundance) from the main text remain, with the 0 Li component being coupled to H (A_{iso} = 8.6 MHz). The weighting of the components did not change, indicating that if this component is present, it still corresponds to our assignment of a surface species. The Li hyperfine coupling constants were allowed to vary but did not change. The fitted hyperfine constant for H corresponds to an NMR shift of 33.9 ppm (using equation 6 in the main text, with g_N = 5.585 for H). To check if this was present, ^{1}H solid-state NMR was performed on the stage 1 sample using a Hahn echo, a pulse sequence typically used to observe hyperfine shifts in NMR, [6,7] spinning at 30 kHz and using a 89 kHz RF strength (Figure S11). No signal was observed in that region, only diamagnetic signals from the electrolyte (ethylene carbonate, 4.5 ppm) and some polyethylene oxide (approx. 6 ppm). Using the 4.5 ppm NMR shift to calculate the corresponding hyperfine coupling constant in EPR produces a value of 2.3 MHz, which does not show in our fits and simulations. We note that the SEI is insulating and diamagnetic (there have been no reports of EPR signals coming from the SEI, even upon *operando* EPR during cycling).[8] The reason why H hyperfine splitting is not as obvious could be linked to the high mobility of protons in the SEI. We also note that if any hyperfine coupling was due to protons in the SEI, this would appear in all spectra and not just those of the dense stages as the SEI is not expected to change with state of charge (lithiation stage).

The fit shown in Figure S10 (e) matches well with the experimental spectrum, expectedly given the increased number of degrees of freedom after including hyperfine to protons. It is possible some hyperfine coupling to protons is occurring but based on our results (EPR and NMR) we believe hyperfine to Li is dominant.

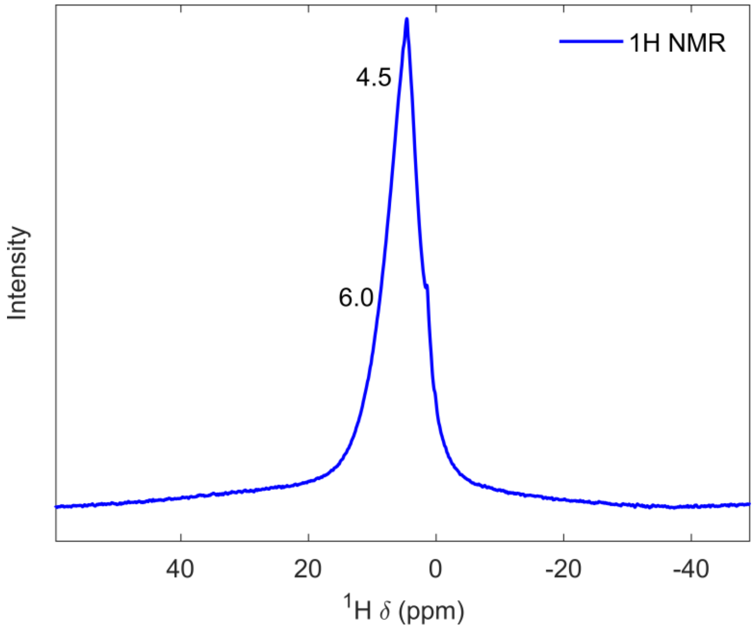

Figure S11. ^{1}H ssNMR spectrum of stage 1 graphite.

12 Simulation at X-band with high-frequency parameters

An X-band spectrum of stage 1 was simulated using the same parameters as the 331 GHz stage 1 spectrum and the phase shift from the X-band spectrum. It shows that the *g*-anisotropy or the hyperfine coupling cannot be resolved at these lower frequencies (as seen experimentally).

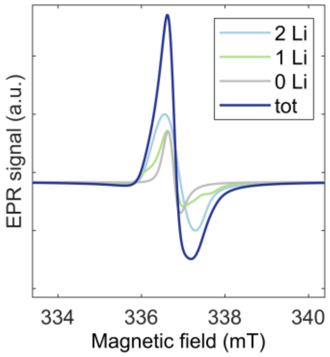

Figure S12. Simulations of stage 1 components at 9.5 GHz (X-band).

13 Fit of the dilute stages

The dilute stages were fitted using a 2-component fit including the 1 Li component and the 0 Li component as described in the main text (Figure 7(c,d)). The lineshapes are in general broader due to faster relaxation rate (65 ns and 50 ns for stages 2L and 4 respectively at 50 K). The fitted hyperfine coupling constants, reported in Table 1 in the main text, match well with the ^{7}Li shifts (at 6.9 ppm A_{iso} = 1.77 MHz, in the fit it is 2.0 MHz; at 4.8 ppm A_{iso} = 0.99 MHz, in the fit it is 1.6 MHz). The error is most likely due to the broader lineshapes observed.

14 Fit at 441GHz of stage 1 sample

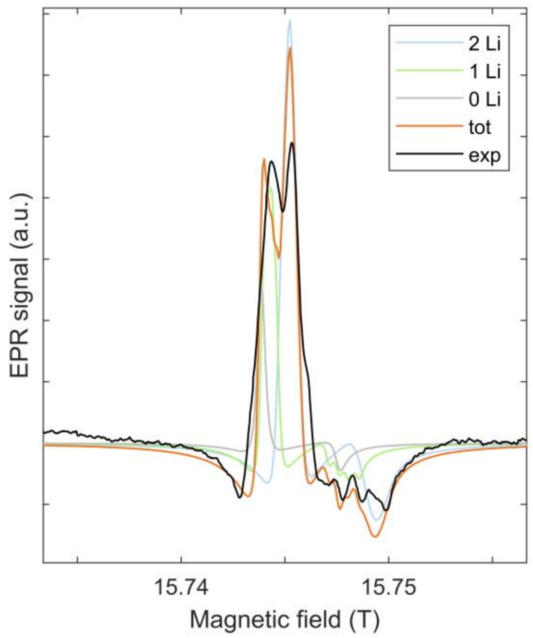

Figure S13. High Frequency EPR spectrum of stage 1 recorded at 441 GHz at 50 K. The fit was performed using the same model as the 331 GHz spectrum shown in Figure 7a.

The shoulder seen on the high field part of this spectrum is also due to the g_z component, as observed in stage 2 and in the dilute stages, but in this case the Easyspin toolkit posed a limit to the goodness of our fit in terms of the phase that can be applied to the different components – Easyspin only allows to set the phase (relevant for samples affected by skin effects) for the entire spectrum, whereas in this case each component has a subtly different phase due to the different degree of metallicity observed (cf. the *A/B* ratios at X-band).

15 Effect of phase on HF fitting

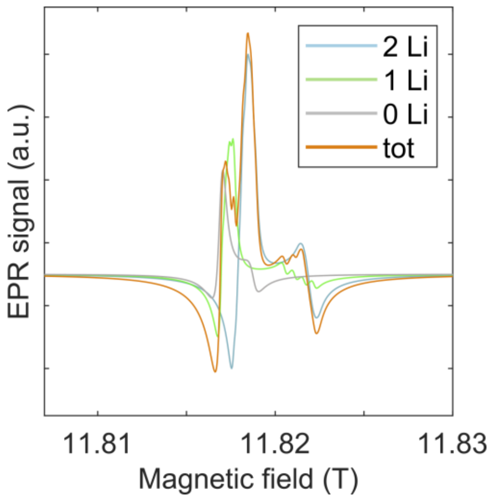

Figure S14. Simulation of the stage 1 fit at 331 GHz (50 K) using the same parameters as in Figure 7a, except for the phase which is set identical to that observed experimentally in the stage 2 spectrum in Figure 7b (phase = 1.5 rad). It can be seen that the components of the fit now look very similar.

Dysonian lineshapes are phase-shifted spectra and the extent of the phase shift is linked to conductivity. Below 180 K, stage 2 becomes more conductive than stage 1, hence explaining the larger phase shift. Here we show how stage 1 would look if the same phase as stage 2 had been applied as we believe it will make comparison across stages easier.

16 Fit of Stage 1 at 10 K and 25 K

To investigate the role of skin effects on the distribution of Li environments in stage 1 in HFEPR, a spectrum taken at 25 K (above its Néel temperature, see Figure S9, 15 K) and one at 10 K of the stage 1 sample were also fitted. Here, the fitting model proposed in the main text (Figure 7) still holds but the weighting of the three different components has changed—the 2 Li component increases to 67% and 72% for 25 K and 10 K respectively (from 59% at 50 K) of the total weight; the 1 Li component decreases to 24% and 19% for 25 K and 10 K respectively (from 31% at 50 K) and the defect spins account for 9% (at both temperatures) of the total weight (from 10% at 50 K). This is consistent with Li^+ intercalation following a concentration gradient, which results in the shell of the particle being more lithiated than the core. It also confirms our assignment of the defect spin component as being confined to the surface of the particles in the dense stages.

By correlating the measured *A/B* ratios to the skin depth and assuming δ = 0.7 μm at 300 K (based on *ab* conductivity by Lauginie and coworkers)[9], and extrapolating *A/B* at 25 and 50 K, based on the slope in Figure S17, it is possible to calculate the skin depth. At 25 K this is approximately 665 nm while at 50 K it is 667 nm, that is δ is shallower at 25 K than at 50 K by approximately 2 nm – it is reasonable to expect that the microwaves are exciting only those microstates towards the surface of the particles (based on particle size of 13 μm, as observed in SEM, Figure S1).

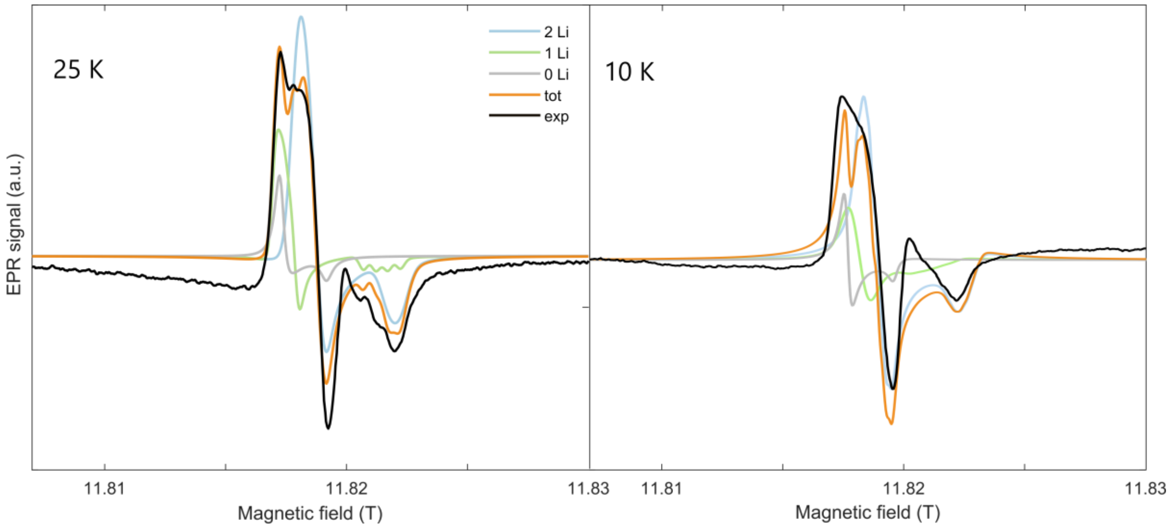

Figure S15. Stage 1 high frequency spectrum collected at 331 GHz (25 K) and related fit with the different components simulated.

The variation in the weights of the two components with temperature for stage 1 at 331 GHz are shown below in Figure S16, in agreement with our conclusion over lithiation gradients within the graphite particles.

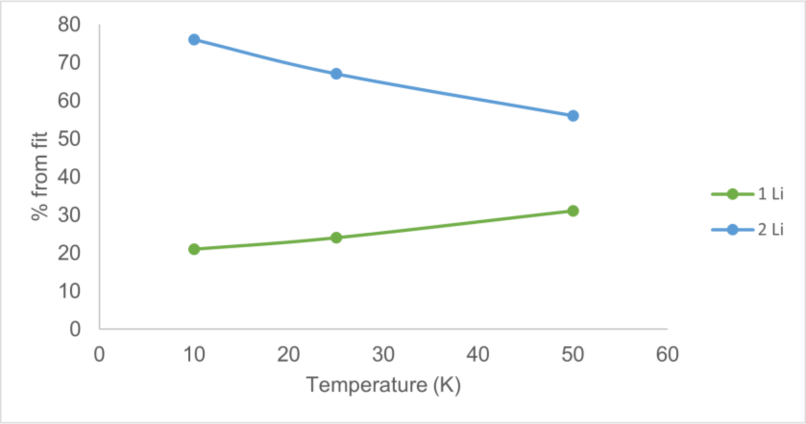

Figure S16. Percentage weight of the 2 Li and 1 Li components for stage 1 at 331 GHz across temperatures. Weights obtained from HFEPR fits.

17 Fits of two regimes of electrical conductivity through *A/B* ratios

The temperature dependence of the *A/B* ratios can be fitted using a linear relationship. It can be seen that two regimes are present between 300-200 K and 200-100 K. The fitting parameters are shown in Table S4, while the fits can be seen in Figure S17. Stage 1 shows a uniform regime across all temperatures (thereby leading to only one slope), while the other stages present two regimes: stage 2 below and above 200 K and stages 2L and 4 below and above 210 K.

Table S4. Fitting parameters for the two different conductivity regimes for the 4 stages.

Stage/ T range		1	2	2L	4
300-200 K	Slope/ K^{-1}	-0.00049	-0.00095	-0.00051	-0.00071
	intercept	2.096	2.18	1.63	1.72
200-100 K	Slope/ K^{-1}	-0.00049	-0.00159	-0.00098	-0.00121
	intercept	2.096	2.30	1.73	1.82

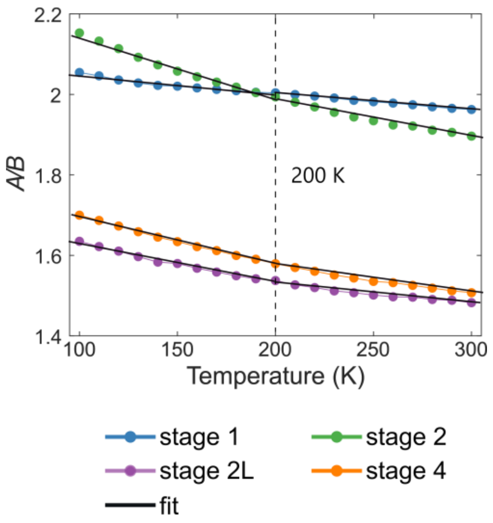

Figure S17. Temperature dependence of the *A/B* ratios for the four stages as seen in the main text. Two conductivity regimes are found between 300-200 K and 200-100 K, as shown in the fits.

18 T_{2e}s derived from the X-band linewidths

Table S5. CW peak-to-peak linewidths (lw) for the 4 stages at 100 K and 300 K as obtained from X-band EPR measurements compared to calculated T_{2e} relaxation times.

Stage/ Temperature	1		2		2L		4	
	T_{2e}/ns	lw/mT	T_{2e}/ns	lw/mT	T_{2e}/ns	lw/mT	T_{2e}/ns	lw/mT
100 K	376.99	0.19	324.68	0.22	360.25	0.20	288.46	0.25
300 K	341.62	0.21	333.17	0.21	377.79	0.19	337.90	0.21

19 Error in fitted Hyperfine coupling constants for stage 1 at 331 GHz

Simulations were carried out where the hyperfine coupling constants for the 2 Li and 1 Li environments was varied by ±5% and ±10%. When the 2 Li component was varied, the 1 Li was kept constant and vice versa. The resulting spectra and total fits are displayed in matrix-like form in the figure below. For the different combinations the new fit (in orange) is also compared to the fit in the main text (grey). For the stage 2 (1 Li) environment, it should be noted that the error in $A_{x,y}$ is larger than A_z due to features being experimentally less resolved; on the other hand, the $A_{x,y}$ components are better defined for the stage 1 (2 Li) environment. In the modelling shown below, it can be seen that varying the 1 Li component produces poorer fits in all scenarios, suggesting the error in A_z is less than 5%, while error in $A_{x,y}$ is larger – in the main text we quote A_{iso}(1 Li) = 3-5 MHz. In the 2 Li component, increasing A reinforces features in the *xy* components but decreasing it completely removes said features producing a much poorer fit, with little change being observed in A_z. This suggests the error in $A_{x,y}$ is <5%, while the error in A_z is approximately 10%.

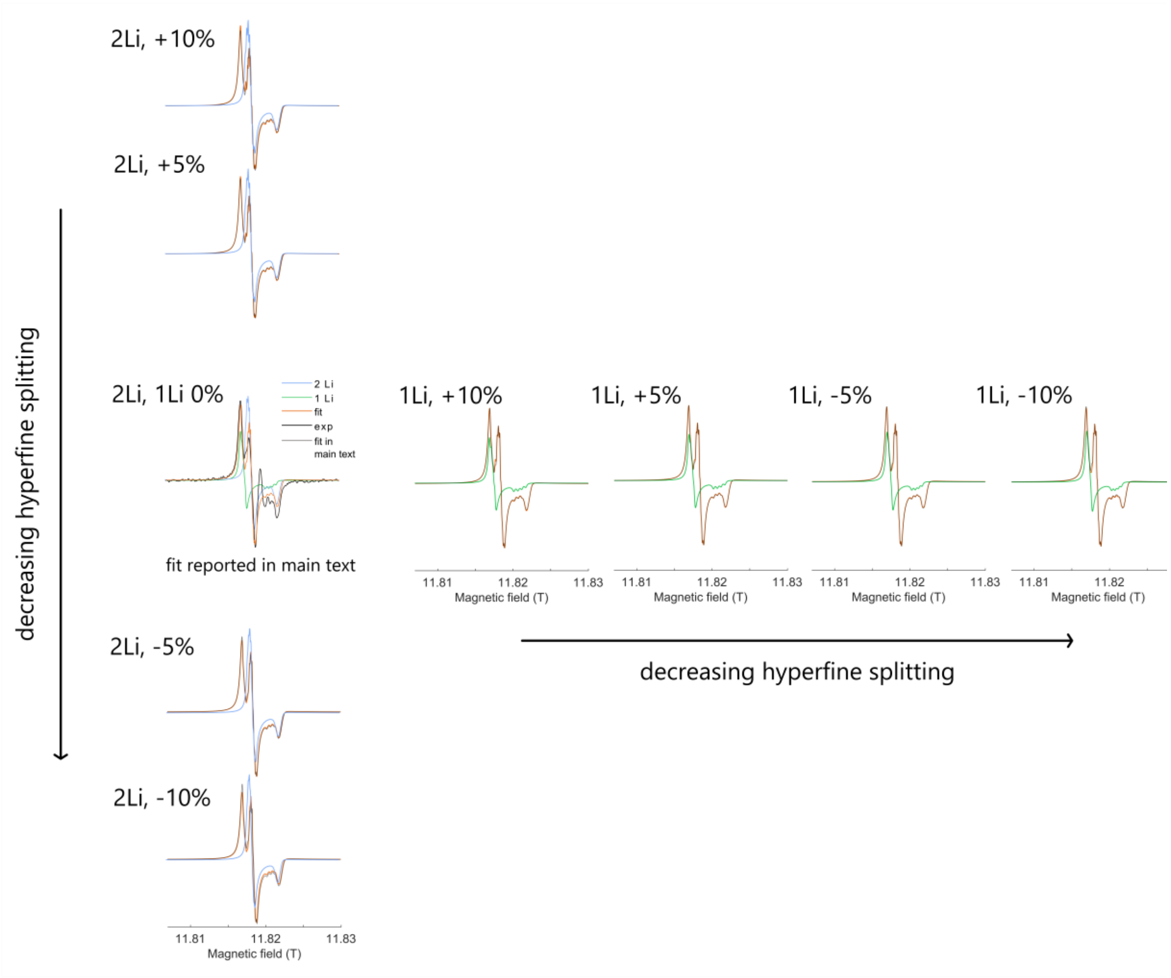

Figure S18. Stage 1 spectrum at 331 GHz recorded at 50 K. The hyperfine coupling constant in the 2 Li (blue) component and 1 Li (green) component are varied from the original fit in the main text (also marked in the figure) by ±5% and ±10%. For the different combinations the new fit (in orange) is also compared to the fit in the main text (grey).

References

(1) Letellier, M.; Chevallier, F.; Morcrette, M. In Situ 7Li Nuclear Magnetic Resonance Observation of the Electrochemical Intercalation of Lithium in Graphite; 1st Cycle. *Carbon* **2007**, *45* (5), 1025–1034. https://doi.org/10.1016/j.carbon.2006.12.018.

(2) Barra, A.-L.; Brunel, L.-C.; Baumann, F.; Schwach, M.; Moscherosch, M.; Kaim, W. High-Frequency (245 GHz) and X-Band EPR Study of Stable Dicopper Radical Complexes. *J. Chem. Soc., Dalton Trans.* **1999**, No. 21, 3855–3857. https://doi.org/10.1039/A903419F.

(3) Wagoner, G. Spin Resonance of Charge Carriers in Graphite. *Phys. Rev.* **1960**, *118* (3), 647–653. https://doi.org/monod.

(4) Singer, L. S.; Wagoner, G. Electron Spin Resonance in Polycrystalline Graphite. *J. Chem. Phys.* **1962**, *37* (8), 1812–1817. https://doi.org/10.1063/1.1733373.

(5) Mukai, K.; Inoue, T. Magnetic Susceptibility Measurements on Li-Intercalated Graphite: Paramagnetic to Diamagnetic Transitions in C12Li Induced by Magnetic Field. *Carbon* **2017**, *123*, 645–650. https://doi.org/10.1016/j.carbon.2017.08.012.

(6) Bassey, E. N.; Reeves, P. J.; Seymour, I. D.; Grey, C. P. 17O NMR Spectroscopy in Lithium-Ion Battery Cathode Materials: Challenges and Interpretation. *J. Am. Chem. Soc.* **2022**. https://doi.org/10.1021/jacs.2c02927.

(7) Märker, K.; Xu, C.; Grey, C. P. Operando NMR of NMC811/Graphite Lithium-Ion Batteries: Structure, Dynamics, and Lithium Metal Deposition. *J. Am. Chem. Soc.* **2020**, *142* (41), 17447–17456. https://doi.org/10.1021/jacs.0c06727.

(8) Wang, B.; Le Fevre, L. W.; Brookfield, A.; McInnes, E. J. L.; Dryfe, R. A. W. Resolution of Lithium Deposition versus Intercalation of Graphite Anodes in Lithium Ion Batteries: An In Situ Electron Paramagnetic Resonance Study. *Angewandte Chemie International Edition* **2021**, *60* (40), 21860–21867. https://doi.org/10.1002/anie.202106178.

(9) Lauginie, P.; Estrade, H.; Conard, J.; Guérard, D.; Lagrange, P.; El Makrini, M. Graphite Lamellar Compounds EPR Studies. *Physica B+C* **1980**, *99* (1), 514–520. https://doi.org/10.1016/0378-4363(80)90288-0.
